# Charge Transport in Solvated Donor–Acceptor Functionalized Peptoids: Molecular Dynamics and Rate Theory

**DOI:** 10.1021/acs.jctc.6c00787

**Published:** 2026-07-23

**Authors:** Zongwei Huang, Bradley S. Harris, Slater T. Bakenhaster, Roshan Khatri, Margaret S. Cheung, Barry D. Dunietz, Marcel D. Baer, Eitan Geva

**Affiliations:** † Department of Chemistry, 1259 University of Michigan, Ann Arbor, Michigan 48109, United States; ‡ 6865 Physical Sciences Division, Pacific Northwest National Laboratory, Richland, Washington 99352, United States; § Department of Chemistry and Biochemistry, 4229 Kent State University, Kent, Ohio 44242, United States

## Abstract

Scalable solar-energy conversion requires photoactive materials that combine the efficiency of natural photosynthetic systems with the stability and processability needed for practical applications. Achieving reliable charge transport in soft, self-assembled organic materials remains challenging, as structural fluctuations and environmental effects strongly influence charge-transfer (CT) rates. Here, we present a broadly applicable computational framework for evaluating CT rates in the condensed phase, combining Fermi’s golden rule rate theory with inputs from all-atom molecular dynamics (MD) simulations and first-principles electronic-structure calculations. The approach does not rely on system-specific parametrization and is applicable to a wide range of soft and disordered materials. We demonstrate the applicability and usefulness of the framework on redox-active peptoids functionalized with iron–porphyrin (Fe–P) complexes, a bioinspired platform with programmable donor–acceptor units and tunable three-dimensional organization. The calculated CT rates exhibit strong sensitivity to molecular conformation, with variations spanning several orders of magnitude. This dependence is shown to arise from the pronounced variation in diabatic electronic coupling with the relative orientations and separations of the Fe–P complexes across the conformational ensemble. The framework provides a consistent route for connecting atomistic structure to CT kinetics in the condensed phase and enables analysis of structure–rate relations in organic semiconducting systems.

## Graphical abstract

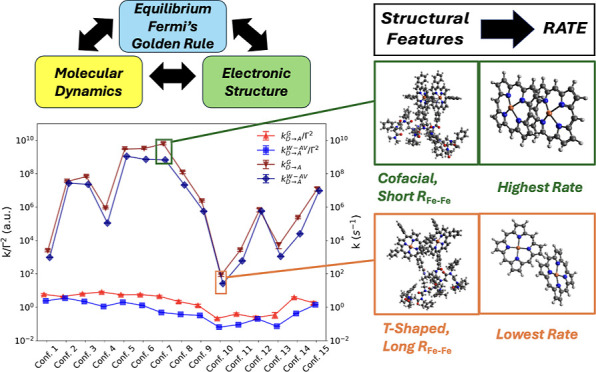

## 1 Introduction

Scalable solar-energy conversion requires photoactive architectures that combine the efficiency of natural photosynthetic systems^1^ with the durability and manufacturability needed for practical devices.^2^ A central challenge is the realization of soft, self-assembled semiconductors that robustly support charge transport (CT).^3^ Addressing this challenge can benefit from computational approaches capable of providing predictive insight into the dependence of CT rates on molecular and electronic structure.^4^ Current approaches, however, face significant limitations, as environmental effects on CT energetics and donor–acceptor electronic coupling are often oversimplified or only approximately treated.^5,6^

In this paper, we introduce a computational framework that links structural degrees of freedom (DOF) to CT rates while explicitly accounting for the underlying molecular and electronic structure.^7^ Achieving this requires an adequate rate theory and ensuring that the potential energy surfaces (PESs) consistently include condensed-phase environmental effects. More specifically, it must quantify the effects of molecular conformation on diabatic couplings between donor and acceptor states,^8^ and the effects of the intramolecular and intermolecular molecular environment in modulating the statistics of the donor–acceptor energy gap.^9^

The utility and feasibility of the proposed framework, which is broadly applicable to soft, self-assembled organic semiconductors, will be demonstrated on a peptoid-based system in liquid solution. Peptoids are *N*-substituted glycine polymers^10^ whose modular side chains enable programmable placement and orientation of donor–acceptor units along a robust backbone, and whose self-assembly into ordered nanostructures can result in well-defined donor–acceptor pathways.^11,12^ In addition, peptoids offer practical advantages, including low-cost synthesis, high yields, biocompatibility, and chemical stability, making them ideal candidates for tunable organic semiconductors.

As shown in (Figure 1top), we focus on a redox-active peptoid chain functionalized with iron–porphyrin (Fe–P) complexes, a bioinspired system with well-defined chemistry that can serve as both donor and acceptor sites in a CT process coupling Fe­(II)–P and Fe­(III)–P states. The system consists of a peptoid with nine glycine units, with Fe–P functionalization at positions 4 and 7. This functionalization enforces relatively short donor–acceptor distances, while the inherent flexibility of the peptoid backbone gives rise to a diverse ensemble of molecular conformations. With the potential for cofacial porphyrin arrangements within helical conformations, the system provides an ideal testbed for methods that correlate molecular geometry with CT rates.

**Figure 1:**
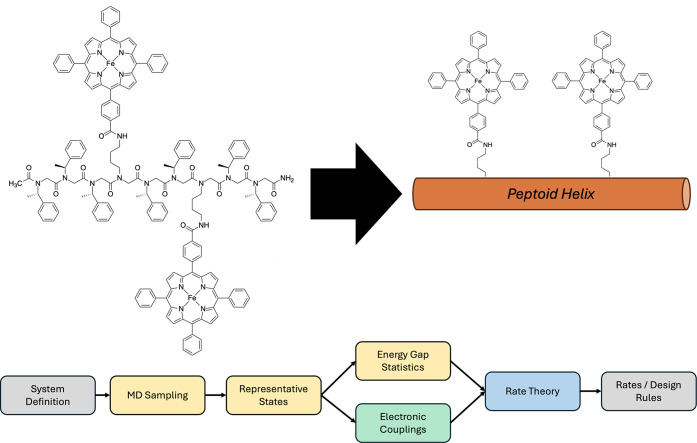
(Top) Schematic of the peptoid model consisting of nine glycine units, with Fe–P functionalization at positions 4 and 7 along the backbone, resulting in a cofacial arrangement of the porphyrin rings. (Bottom) Computational workflow illustrating the integration of MD simulations, electronic structure calculations, and rate theory for predicting condensed-phase CT rates between the Fe–P units.

Our computational framework integrates condensed-phase electronic structure calculations with molecular dynamics (MD)-based energy-gap statistics to compute CT rates within the equilibrium Fermi’s golden rule (E-FGR) framework. This is achieved either by mapping to the spin-boson model or by employing the linearized semiclassical (LSC) approximation.^13^ We consider a systematically derived hierarchy of approximate E-FGR rate expressions, including a Marcus-like rate expression as the most approximate form, which serves as a benchmark for validation (see Figure 1bottom) for illustrating the overall workflow.

Our rate calculations are based on inputs from density functional theory (DFT) calculations and all-atom MD simulations and account for the effect of the wide range of conformations and solvent configurations on the CT rates.^14^ The framework begins by defining the peptoid backbone, functionalization sites, and donor and acceptor units, and sampling their relative orientations to establish representative donor–acceptor pairs. For this set, donor–acceptor energy-gap correlation functions^15^ and diabatic couplings^16^ are obtained from MD simulations and first-principles calculations, respectively.^15–17^ These inputs are then used within the E-FGR framework to obtain CT rates, providing a direct link between molecular geometry, solvation dynamics, and CT rates.^13,18–20^

The rest of this paper is organized as follows. The computational framework is described in Section 2, with the rate theory detailed in Subsection [Sec sec2.1], the MD simulations in Subsection [Sec sec2.2], and the electronic coupling calculations in Subsection [Sec sec2.3]. Results from MD sampling and the computed CT rates are presented and discussed in Section 3, followed by concluding remarks and a summary of the main results in Section 4. Technical details and data are provided in the Appendices and Supporting Information.

## 2 Computational Framework

Our computational approach integrates three key components: (1) all-atom molecular dynamics (MD) simulations to sample donor–acceptor conformational space and compute solvent-induced energy-gap fluctuations; (2) first-principles electronic structure calculations to determine diabatic couplings in solution via constrained DFT (CDFT); and (3) rate theory based on E-FGR,^5,21–24^ employing both the LSC^13,25–27^ approximation and mapping onto the spin–boson Hamiltonian.^28–37^

The overall computational workflow is illustrated in the bottom inset of Figure 1. It begins with system setup and the generation of a representative ensemble of peptoid conformations functionalized with a pair of Fe–porphyrin units. For each conformation, MD simulations are performed to obtain the statistics of donor–acceptor energy-gap fluctuations. In addition, diabatic electronic couplings between the Fe–P complexes are calculated via condensed-phase electronic structure calculations. This is followed by calculating a hierarchy of E-FGR CT rate constants based on the energy-gap statistics and the electronic couplings. A more detailed description of the components of this workflow are provided in the following subsections.

### 2.1 Rate Theory

#### 2.1.1 E-FGR CT Rate Constants

We begin with the CT rate theory. To this end, we start out with the generic two-state Hamiltonian typically used to describe CT between a donor state, *D*, and an acceptor state, *A* (in what follows, boldfaced variables, e.g., **V**, represent vector quantities, and a hat over a variable, e.g., Ô, denotes an operator)

1 $$Ĥ={Ĥ}_{D}|D\rangle \langle D|+{Ĥ}_{A}|A\rangle \langle A|+\Gamma \left(|D\rangle \langle A\left|+|A\rangle \langle D\right|\right)$$

Here, |*D*⟩ and |*A*⟩ denote the diabatic donor and acceptor states, respectively. ${Ĥ}_{D}$ and ${Ĥ}_{A}$ are the nuclear Hamiltonians for the system in the donor and acceptor states, respectively, given by ${Ĥ}_{D/A}=\frac{{\mathrm{P̂}}^{2}}{2}+V_{D/A}\left(\mathrm{R̂}\right)$ where $\mathrm{R̂}=\left({\mathrm{R̂}}_{1},{\mathrm{R̂}}_{2},...,{\mathrm{R̂}}_{N}\right)$ and $\mathrm{P̂}=\left({\mathrm{P̂}}_{1},{\mathrm{P̂}}_{2},...,{\mathrm{P̂}}_{N}\right)$ are the *N* ≫ 1 mass-weighted coordinates and momenta of the nuclear DOF. The functions $V_{D/A}\left(\mathrm{R̂}\right)$ are the donor and acceptor PESs, and Γ is the donor–acceptor diabatic electronic coupling coefficient within the Condon approximation. We adopt the convention that the D and A states are designated such that *D* → *A* corresponds to a *downhill* CT reaction, and *A* → *D* corresponds to an *uphill* CT reaction.

The initial state of the overall system (nuclear + electronic DOFs) is assumed to be given by a density operator of the following form

2 $$\mathrm{\rho ̂}\left(0\right)={\mathrm{\rho ̂}}_{j}^{eq}|j\rangle \langle j|,{\mathrm{\rho ̂}}_{j}^{eq}=e^{-{Ĥ}_{j}/k_{B}T}/{Tr}_{n}\left[e^{-{Ĥ}_{j}/k_{B}T}\right]$$

Here, *j* = *D*, *A*, *T*r_*n*_ is the trace over the nuclear Hilbert space, *k*_*B*_ is the Boltzmann constant, and *T* is the absolute temperature. This implies that the nuclear DOF are initially in thermal equilibrium with respect to the initial electronic state (donor or acceptor).

Given the initial state in eq 2 and treating the diabatic electronic coupling term in eq 1, Γ­(|*D*⟩⟨*A*| + |*A*⟩⟨*D*|), as a small perturbation, the CT rate constants, *k*_*j*→*k*_, within second-order time-dependent perturbation theory, are given by the following E-FGR expression^25^

3 $$k_{j\to k}=\frac{2}{\hbar^{2}}Re\int_{0}^{\infty }dt,C_{j\to k}\left(t\right)$$

where

4 $$C_{j\to k}\left(t\right)=\Gamma^{2}{Tr}_{n}\left[{\mathrm{\rho ̂}}_{j}^{eq}e^{i{Ĥ}_{j}t/\hbar }e^{-i{Ĥ}_{k}t/\hbar }\right]$$

here, *j* = *D*, *A* and *k*_*j*→*k*_ is the E-FGR rate constant for the *j* → *k* CT reaction (*j* ≠ *k*). In particular, *k*_*D*→*A*_ and *k*_*A*→*D*_ correspond to the downhill and uphill CT rates, respectively. The description in terms of rate constants is only valid on time scales longer than the lifetime of the correlation function *C*_*j*→*k*_(*t*). Such separation of time scales holds for the systems considered here as the lifetime of *C*_*j*→*k*_(*t*) is on the femtosecond time scale, and therefore much shorter than $k_{j\to k}^{-1}$ .

The E-FGR expression for the CT rate constant in eq 3 is fully quantum-mechanical, in that evaluating *C*_*j*→*k*_(*t*) in eq 4 requires simulating the quantum dynamics of both electronic and nuclear DOF. Such a simulation is not feasible for the all-atom model Hamiltonian of the peptoid-based system considered here. Accordingly, two approximations are employed for calculating *C*_*j*→*k*_(*t*) and the resulting *k*_*j*→*k*_:

1. LSC, which is outlined in Section 2.1.2.^25–27^

2. Mapping to a spin-boson Hamiltonian, which is outlined in Section 2.1.3.^13^

The two approximate methods provide complementary perspectives on CT dynamics. Mapping onto a spin-boson model enables the calculation of fully quantum-mechanical E-FGR rate constants and provides a practical route for estimating dynamical and quantum effects, but also requires assuming that the donor and acceptor PESs are identical except for a shift in equilibrium energy and geometry. In contrast, the LSC approximation does not impose this restriction, but is only computationally feasible for the all-atom model in the case where the CT rate constant is insensitive to dynamical and quantum effects and the donor–acceptor energy-gap fluctuations follow *classical Gaussian statistics*.

#### 2.1.2 The LSC Approximation

A fully quantum-mechanical closed-form expression for *C*_*j*→*k*_(*t*) in eq 4 can only be derived when the diabatic PESs are assumed to be harmonic (see Section 2.1.3).^21,28,38–43^ The LSC approximation outlined in this subsection provides an approximate practical route for calculating *C*_*j*→*k*_(*t*) which is applicable to an all-atom model Hamiltonian based on anharmonic force fields.

The derivation of the LSC approximation begins by writing *C*_*j*→*k*_(*t*) in real-time Feynman path integral form,^44–46^ followed by expanding the forward–backward action to first order with respect to the difference between the forward and backward paths.^9,25,47^ The resulting LSC approximation for *C*_*j*→*k*_(*t*) is then given by

5 $$\begin{array}{ll} C_{j\to k}^{W‐AV}\left(t\right) & \\ = & \Gamma^{2}\int dR_{0}\int dP_{0}{\left[{\mathrm{\rho ̂}}_{j}^{eq}\right]}_{W}\left(R_{0},P_{0}\right)e^{i\int_{0}^{t}dt^{\prime }U_{jk}\left(R_{t^{\prime }}\right)/\hbar }\\ \equiv & \Gamma^{2}{\left\langle e^{i\int_{0}^{t}dt^{\prime }U_{jk}\left(R_{t^{\prime }}\right)/\hbar }\right\rangle }_{j}^{W,eq} \end{array}$$

here, *U*_*jk*_(**R**) = *V*_*j*_(**R**) – *V*_*k*_(**R**) is the difference between the initial and final states’ PESs, ${\left[{\mathrm{\rho ̂}}_{j}^{eq}\right]}_{W}\left(R_{0},P_{0}\right)$ is the Wigner transform^48^ of ${\mathrm{\rho ̂}}_{j}^{eq}$ , and **R**_*t*′_ is obtained via classical dynamics, starting at {**R**_0_, **P**_0_} and propagating on the average PES, *V*_av_(**R**) = [*V*_*j*_(**R**) + *V*_*k*_(**R**)]/2. The “W-AV” label stands for Wigner (W) initial sampling (based on quantum thermal equilibrium on the *j*-th PES) with dynamics on the average (AV) PES. We also note that despite its classical-like nature, the W-AV approximation yields the *exact* quantum-mechanical E-FGR CT rate constant when the *j* and *k* PESs are parabolic and identical except for a shift in equilibrium energy and equilibrium geometry (i.e., the spin-boson model; see Section 2.1.3).^26^

Calculating the CT rate constant based on eq 5 in an all-atom model is still highly challenging, since it requires sampling based on the Wigner distribution and averaging over a large number of costly *nonequilibrium* MD simulations. One possible simplification involves replacing Wigner sampling with classical sampling and performing the dynamics on the *j*-th PES instead of the average PES. Making those simplifications leads to an approximation that we label “C-j,” which stands for classical (C) initial sampling with dynamics on the *j*-th PES. Further simplification is based on the fact that ${\left\langle e^{i\int_{0}^{t}d\tau^{\prime }U\left(R_{\tau^{\prime }}\right)/\hbar }\right\rangle }_{j,Cl}^{eq}$ is often observed to decay to zero faster than the nuclear dynamics time scale on which *U*(**R**_τ′_) changes, which leads to an approximation that can be calculated via an *equilibrium* MD simulation

$$\begin{array}{lll} C_{j\to k}^{C‐0}\left(t\right) & = & \Gamma^{2}\int dR_{0}\int dP_{0}{\left[{\mathrm{\rho ̂}}_{j}^{eq}\right]}_{Cl}\left(R_{0},P_{0}\right)e^{iU_{jk}\left(R_{0}\right)t/\hbar }\\ & \equiv & \Gamma^{2}{\left\langle e^{iU\left(R_{0}\right)t/\hbar }\right\rangle }_{j}^{Cl,eq} \end{array}$$ 6

the label “C-0” stands for classical (C) initial sampling on the *j*-th PES, combined with lack of dependence on the nuclear dynamics (0).

Even with the aforementioned simplifications, calculating the LSC approximation for an all-atom model can still be computationally challenging. This is because the oscillatory nature of the quantity averaged over makes it difficult to converge the average. One way of bypassing this problem is by assuming Gaussian statistics, which allows one to replace ${\left\langle e^{iU\left(R_{0}\right)t/\hbar }\right\rangle }_{j}^{Cl,eq}$ in eq 6 with the corresponding second-order cumulant approximation

7 $$C_{j\to k}^{G}\left(t\right)=\Gamma^{2}e^{i{\left\langle U_{jk}\right\rangle }_{j}^{Cl,eq}t/\hbar -\sigma_{jk}^{2}t^{2}/2\hbar^{2}}$$

here

8 $$\sigma_{jk}^{2}={\left\langle U_{jk}^{2}\right\rangle }_{j}^{Cl,eq}-{\left({\left\langle U_{jk}\right\rangle }_{j}^{Cl,eq}\right)}^{2}$$

is the variance of *U*_*jk*_ at equilibrium on the *j*-th PES. The label *G* denotes the Gaussian-statistics (G) approximation. For simplicity of notation, in what follows we let ⟨···⟩_*j*_ represent ${\left\langle \cdot \cdot \cdot \right\rangle }_{j}^{Cl,eq}$ . Because $C_{j\to k}^{G}\left(t\right)$ is Gaussian in time, the integral in eq 3 can be evaluated in closed form, yielding the following approximate E-FGR rate constant

9 $$k_{j\to k}^{G}=\frac{1}{\hbar }\Gamma^{2}\sqrt{\frac{2\pi }{\sigma_{jk}^{2}}}\exp\left[-\frac{{\left\langle U_{jk}\right\rangle }_{j}^{2}}{2\sigma_{jk}^{2}}\right]$$

Given the high computational cost of evaluating the higher-level approximations in the hierarchy [ $C_{j\to k}^{W‐AV}\left(t\right)$ , $C_{j\to k}^{C‐AV}\left(t\right)$ , $C_{j\to k}^{C‐D}\left(t\right)$ and $C_{j\to k}^{C‐0}\left(t\right)$ ] for an all-atom model, we restrict these calculations to the spin-boson model onto which the all-atom system is mapped (see Section 2.1.3). The rate constants calculated for the spin-boson model are then used to assess the role of quantum and dynamical effects in determining CT rates. For the peptoid system under consideration, those calculations show that dynamical and quantum effects on the CT rate constants are negligible. This justifies calculating the CT rate constants for the all-atom model within the Gaussian-statistics expression in eq 9. To this end, we performed calculations of both donor-to-acceptor and acceptor-to-donor rate constants, based on eq 9

10 $$\begin{array}{ll} k_{D\to A}^{G} & =\frac{\Gamma^{2}}{\hbar }\sqrt{\frac{2\pi }{\sigma_{DA}^{2}}}\exp\left[-\frac{{\left\langle U_{DA}\right\rangle }_{D}^{2}}{2\sigma_{DA}^{2}}\right],\\ k_{A\to D}^{G} & =\frac{\Gamma^{2}}{\hbar }\sqrt{\frac{2\pi }{\sigma_{AD}^{2}}}\exp\left[-\frac{{\left\langle U_{AD}\right\rangle }_{A}^{2}}{2\sigma_{AD}^{2}}\right] \end{array}$$

importantly, ${\left\langle U_{DA}\right\rangle }_{D}$ and σ_*DA*_^2^ are obtained from sampling on the *donor* PES, while ${\left\langle U_{AD}\right\rangle }_{A}$ and σ_*AD*_^2^ are obtained from sampling on the *acceptor* PES.

The reaction free energy, Δ*E*_*D*→*A*_, can be obtained from the detailed balance relation

11 $$\Delta E_{D\to A}=-k_{B}T\;\ln\left(\frac{k_{A\to D}^{G}}{k_{D\to A}^{G}}\right)=\left[\frac{{\left\langle U\right\rangle }_{D}^{2}}{2\sigma_{U_{D}}^{2}}-\frac{{\left\langle U\right\rangle }_{A}^{2}}{2\sigma_{U_{A}}^{2}}\right]k_{B}T$$

this provides an unambiguous definition of the reaction free energy that satisfies microscopic reversibility (Δ*E*_*D*→*A*_ = −Δ*E*_*A*→*D*_), a property that is particularly important for systems in which the CT reaction is slightly asymmetric, such as the peptoid systems studied here. Although eq 10 is based on assumptions similar to those underlying standard Marcus theory, it is not equivalent to the Marcus expression; a more detailed discussion of both points is provided in Appendix A.

#### 2.1.3 Mapping to the Spin-Boson Hamiltonian

A widely used approach for simulating quantum dynamics in the condensed phase is based on mapping the all-atom Hamiltonian onto the spin-boson Hamiltonian^6,28,29^

12 $$Ĥ=\Gamma {\mathrm{\sigma ̂}}_{x}-\frac{\Delta E}{2}{\mathrm{\sigma ̂}}_{z}+\sum_{j=1}^{N}\left(\frac{{\mathrm{p̂}}_{j}^{2}}{2}+\frac{1}{2}\omega_{j}^{2}{\mathrm{x̂}}_{j}^{2}-c_{j}{\mathrm{x̂}}_{j}{\mathrm{\sigma ̂}}_{z}\right)$$

here, ${\mathrm{\sigma ̂}}_{x}=|D\rangle \langle A|+|A\rangle \langle D|$ , ${\mathrm{\sigma ̂}}_{z}=|D\rangle \langle D|-|A\rangle \langle A|$ , Γ is the diabatic electronic coupling coefficient (see eq 1), Δ*E* ≡ Δ*E*_*D*→*A*_ ≤ 0 is the donor-to-acceptor reaction free energy (see eq 11), $\left\{{\mathrm{x̂}}_{j},{\mathrm{p̂}}_{j},\omega_{j},c_{j}|j=1,...,N\right\}$ are the mass-weighted normal modes’ coordinates and momenta, frequencies and coupling coefficients between the electronic DOF and normal modes, respectively.

It should be noted that the spin-boson Hamiltonian can be cast in a form equivalent to that of the Hamiltonian in eq 1, with ${Ĥ}_{D}$ and ${Ĥ}_{A}$ given by

13 $$\begin{array}{lll} {Ĥ}_{D} & = & \sum_{j=1}^{N}\frac{{\mathrm{p̂}}_{j}^{2}}{2}+\frac{1}{2}\sum_{j=1}^{N}\omega_{j}^{2}{\mathrm{x̂}}_{j}^{2}\\ {Ĥ}_{A} & = & \sum_{j=1}^{N}\frac{{\mathrm{p̂}}_{j}^{2}}{2}+\Delta E+\frac{1}{2}\sum_{j=1}^{N}\omega_{j}^{2}{\left({\mathrm{x̂}}_{j}-x_{j}^{eq}\right)}^{2}. \end{array}$$

here, *x*_*j*_^eq^ = 2*c*_*j*_/ω_*j*_^2^ is the shift in equilibrium geometry between the donor and acceptor PESs along the *j*-th normal mode coordinate. The donor and acceptor PESs within the spin-boson model are harmonic and differ only through shifts in equilibrium energy and equilibrium geometry (Δ*E* and {*x*_*j*_^eq^|*j* = 1,...*N*}, respectively).

Specific realizations of the spin-boson model are determined by the values of {ω_*j*_, *c*_*j*_|*j* = 1, ..., *N*}, which can in turn be obtained from the *spectral density* function

14 $$J\left(\omega \right)=\frac{\pi }{2}\sum_{j=1}^{N}\frac{c_{j}^{2}}{\omega_{j}}\delta \left(\omega -\omega_{j}\right)$$

the procedure employed in this paper for mapping an all-atom Hamiltonian like that of the peptoid system onto the spin-boson Hamiltonian is based on the following identity^6^

15 $$J\left(\omega \right)=\beta \omega \int_{0}^{\infty }dtC_{UU}\left(t\right)\cos\left(\omega t\right)$$

in eq 15, *C*_*UU*_(*t*), is a *classical* equilibrium two-time autocorrelation function, obtainable from classical MD simulations of the all-atom model

16 $$C_{UU}\left(t\right)=\left\langle U\left(0\right)U\left(t\right)\right\rangle -\langle {U\rangle }^{2}$$

here, *U*(**R**) is the energy gap between the donor and acceptor PESs at the nuclear configuration **R**

17 $$U\left(R\right)\equiv U_{DA}\left(R\right)=V_{D}\left(R\right)-V_{A}\left(R\right)$$

such that *U*(*t*) = *U*(**R**_*t*_), where **R**_*t*_ is the nuclear configuration at time *t* obtained from an *equilibrium classical MD simulation* on the donor PES, *V*_*D*_(**R**). In eq 16 ⟨···⟩ corresponds to an average over trajectories whose initial states are sampled from the *classical* Boltzmann distribution $e^{-H_{D}\left(R,P\right)/k_{B}T}/Q_{D}$ . Computational details on the calculation of *C*_*UU*_(*t*) from MD inputs are provided in Appendix B. The discretization of the continuous spectral density function *J*(ω) (see eq 15) was based on a procedure that was adopted from ref 29. Finally, the rate constant for the backward acceptor-to-donor reaction, *k*_*A*→*D*_, was obtained from the forward donor-to-acceptor rate constant, *k*_*D*→*A*_, via detailed balance: $k_{A\to D}=k_{D\to A}e^{\Delta E/k_{B}T}$ , where Δ*E* ≡ Δ*E*_*D*→*A*_ is as in eq 11.

Once the all-atom Hamiltonian is mapped onto a spin-boson Hamiltonian, the *quantum-mechanically exact* correlation function *C*_*D*→*A*_(*t*) can be obtained in closed form^26^

18 $$C_{D\to A}\left(t\right)\Gamma^{2}=\exp\left\{-\frac{i}{\hbar }\Delta Et-\sum_{j=1}^{N}\frac{\omega_{j}{\left(R_{j}^{eq}\right)}^{2}}{2\hbar }\times \left[\coth\left(\frac{\hbar \omega_{j}}{2k_{B}T}\right)\left(1-\cos\left(\omega_{j}t\right)\right)+i\;\sin\left(\omega_{j}t\right)\right]\right\}$$

for the spin-boson model, eq 18 coincides exactly with the LSC approximation $C_{D\to A}^{W-AV}\left(t\right)$ in eq 5.^26^

Within the spin-boson framework, closed form expressions can also be obtained for the hierarchy of approximate expressions for *C*_*D*→*A*_(*t*) described in Section 2.1.2 (see Appendix C). Unlike for the all-atom model, the fact that the entire hierarchy of approximations is given in closed form in the case of the spin-boson model, makes their calculation feasible.

### 2.2 MD Simulations

We investigate CT between donor–acceptor (*D*–*A*) sites composed of iron–porphyrin (Fe–P) complexes. Specifically, we consider a pair of Fe–P units functionalized onto a peptoid backbone of nine glycine units, at positions 4 and 7, as illustrated in (Figure 1 Top). Functionalization at these central positions results in a relatively short *D*–*A* separation, enabled by the conformational flexibility of the peptoid backbone, and therefore the potential for sufficiently strong electronic coupling between the porphyrin units. The solvent is acetonitrile, a polar medium that facilitates charge separation and presents a dielectric environment similar to that encountered in many solution-phase and biomimetic electron-transfer systems.

A representative ensemble of peptoid–porphyrin complex conformations was generated to capture the relative alignment of the donor and acceptor porphyrin units. Long-time scale equilibrium MD simulations were first used to sample the conformational ensemble of the solvated complex. The representative bound donor–acceptor (*D*–*A*) configurations were then selected from this ensemble for follow-up analysis. For each selected fixed nuclear configuration, 200 independent MD simulations (100 ps each) with randomized initial solvent velocities were performed to sample solvent reorganization dynamics at fixed *D*–*A* geometry. The resulting solvent-induced fluctuations were used to compute energy-gap averages and correlation functions, which provide the input for calculating CT rate constants (see Section 2.1).

#### 2.2.1 System Preparation and Equilibrium Sampling

Initial Fe–P-containing peptoid structures were built using tleap in AmberTools with the STEPs force field for peptoids.^49,50^ Side chains corresponding to Fe­(II) and Fe­(III) tetraphenylporphyrins (TPPs) with a long linker, analogous to those studied by Kang et al., were added to the STEPs force field using the three-letter residue code HEM.^51^ Charges for these side chains were generated as described in the original STEPs publication, using NWCHEM to obtain RESP charges for both Fe­(II) and Fe­(III).^49,52^ To incorporate the Fe atoms into the STEPs force field, parameters based on the HEME group in the MCPB.py tutorial were added via an FRCMOD file, and atom types for the iron and coordinating nitrogens were modified to accommodate these custom parameters.^53^ All MOL2 files, FRCMODs, and tleap input files are available in the Supporting Information.

The peptoid backbone with Fe–TPP side chains was built based on the helix reported by Kang et al., with 6 Å spacing between TPP side chains.^51^ The initial structures were assumed to adopt an ideal helical geometry based on electronic circular dichroism (ECD), and were built accordingly using tleap. Following structure generation, the initial coordinates and topology were converted from Amber to GROMACS format using ParmEd.^50,54^ The resulting systems were energy-minimized via steepest descent and solvated with 2152 acetonitrile molecules.^55^

MD simulations were performed using Gromacs 2020.6.^54^ After energy minimization, the solvated structures underwent a brief 50 ps initialization consisting of velocity generation and pressure relaxation in the *NPT* ensemble using the Berendsen thermostat at 300 K and Berendsen barostat at 1 bar.^56^ Production simulations were then performed for 430 ns in the *NPT* ensemble at 300 K and 1 bar using the velocity-rescale thermostat and Parrinello–Rahman barostat.^57,58^ Simulations employed a 1 fs time step, the Verlet integrator, 1.2 nm electrostatic and van der Waals cutoffs, and particle-mesh Ewald for long-range electrostatics.^59^ Temperature coupling was applied independently to solvent and peptoid groups with a 0.1 ps time constant, and pressure coupling was handled isotropically with a 2 ps time constant. Position restraints were applied to the backbone nitrogens during production to maintain the assumed helical structure based on literature while allowing the side-chains to sample freely.^51,60^ All production simulation files are included in the SI to facilitate reproducibility.

#### 2.2.2 Selection of Representative Conformations

Following the production simulations, the trajectories were wrapped and centered to account for periodic boundary conditions, and the Fe–Fe distances between the porphyrin rings were calculated. These distances were used to identify potential bound (Fe–Fe distance <11 Å) and unbound states with a custom TCL script in VMD (available in the SI).^61,62^ To reduce bias from the idealized starting geometry, structures were selected from bound configurations sampled after the first unbinding event. A set of 15 conformations was chosen to span the range of observed *D*–*A* separations and relative porphyrin-ring orientations and was used for subsequent electronic-structure calculations and short fixed-conformation solvent simulations.

#### 2.2.3 Fixed-Conformation Solvent Sampling for Energy-Gap Statistics

For each selected structure, velocities were generated via 10 ps NVT simulations using the velocity-rescale thermostat, with temperature coupling applied separately to the peptoid and solvent groups.^57^ Two hundred independent sets of velocities per structure were created using different random seeds. Each set was then used to initiate a 100 ps NVE production simulation with the peptoid conformation frozen (using the Gromacs freezegrps option), a 1 fs time step, and 1 fs trajectory writeout.^54^ This protocol isolates solvent-induced energy-gap fluctuations at fixed solute geometry.

Full-resolution trajectories were subsequently processed using the gmx rerun feature. A second topology was constructed by swapping the iron charge localization to the opposite porphyrin site (bonded/nonbonded parameters untouched), and energy differences between the original and swapped topologies were evaluated along each trajectory.^54^ These energy-gap fluctuations were then used to construct the averages and correlation functions required for the calculations of CT rate constants (see Section 2.1).

### 2.3 Condensed Phase Electronic Structure

Electronic couplings for the rate calculations are obtained using diabatic charge-constrained DFT with solvent effects included by dielectric screened range-separated hybrid (SRSH) functional combined with a polarizable continuum model (PCM).^63–71^ SRSH functionals are based on a generalized Kohn–Sham (GKS) formalism,^72–75^ where the Coulomb operator is partitioned into short- and long-range (SR and LR) contributions^76–89^

19 $$\frac{1}{r}=\frac{\alpha +\beta \mathrm{erf}\left(\omega r\right)}{r}+\frac{1-\left(\alpha +\beta \mathrm{erf}\left(\omega r\right)\right)}{r}$$

yielding a GKS exchange–correlation energy expression of the following form

20 $$\begin{array}{l} E_{XC}^{GKS}=\alpha E_{F_{X}}^{SR}+\left(1-\alpha \right)E_{DF_{X}}^{SR}+\left(\alpha +\beta \right)E_{F_{X}}^{LR}\\ +\left(1-\alpha -\beta \right)E_{DF_{X}}^{LR}+E_{DF_{C}} \end{array}$$

here *X* and *C* denote exchange and correlation, F and DF denote Fock and DFT-based exchange, and ω is the range-separation parameter optimized by minimizing deviations of frontier orbital energies from ionization potentials. In the gas phase, α + β = 1 ensuring the correct unscreened LR Coulomb interaction limit. In polarizable condensed-phase environments, the LR limit is redefined to impose the environmental dielectric screening, parametrized by the dielectric constant (ε), where α + β = 1/ε, establishing the SRSH functionals.^27,86,90–92^ SRSH functionals, in combination with a polarizable continuum model (SRSH-PCM), have been successfully benchmarked for describing the electronic structure of solvated molecular systems.^27,86,90–97^

Implementing the SRSH-PCM framework described above proceeds by first selecting a value of α for the SR exact exchange and then redefining β to account for the solvent environment according to

21 β=1/ε−α

as reported previously, invoking PCM modifies the ionization potential (IP) and electron affinity (EA), but in standard RSH-PCM calculations without dielectric screening within the functional, the frontier orbital energies remain close to their gas-phase values, leading to misalignment with the IP and EA. The SRSH-PCM framework, which explicitly incorporates dielectric screening in the LR exchange, ensures proper alignment of the Frontier orbital energies with the IP and EA.^87^

The electronic coupling between the donor and acceptor diabatic states is obtained using charge-constrained DFT (CDFT).^98,99^ In CDFT, self-consistent calculations are performed under imposed constraints on the charge or spin of the designated donor and acceptor sites.^100^ The diabatic states are used to form the active configuration-interaction space

22 $$\left(\begin{array}{ll} H_{11} & H_{12}\\ H_{21} & H_{22} \end{array}\right)\left(\begin{array}{l} c_{1}\\ c_{2} \end{array}\right)=E\left(\begin{array}{ll} 1 & S_{12}\\ S_{21} & 1 \end{array}\right)\left(\begin{array}{l} c_{1}\\ c_{2} \end{array}\right)$$

where *H* is the Hamiltonian, *c*_1_ and *c*_2_ are the coefficients, and *S* is the overlap matrix.^101^ The off-diagonal *H* elements correspond to the electronic couplings between the two states. The reported couplings are obtained in an orthogonalized basis.^99,101,102^

In calculating the electronic couplings, unless otherwise noted, the porphyrin rings retain the intermolecular orientations in the sampled MD trajectory snapshots, and where the solvent molecules are replaced by a PCM representing acetonitrile. For each structure, the CDFT-CI calculation was performed using the SRSH-PCM approach with a 6–31G* basis set, α = 0.2, ω = 0.175, and ε = 37.5 that corresponds to the scalar dielectric constant of acetonitrile addressed as the solvent. The ω value was chosen through optimally tuning for the range-separation in the gas phase. All electronic structure calculations were carried out using Q-Chem 6 software package.^103^

### 2.4 Summary of the Computational Workflow

In summary, the key steps of the computational workflow are as follows:

1. Conformation Selection: Fifteen representative conformations were chosen based on MD simulations.
2. Energy-gap Statistics: For each conformation, 200 independent 100 ps trajectories were generated and used to sample solvent dynamics and calculate ${\left\langle U_{DA}\right\rangle }_{D}$ , ${\left\langle U_{AD}\right\rangle }_{A}$ , σ_*DA*_^2^, σ_*AD*_^2^, and *C*_*UU*_(*t*).
3. Electronic State Coupling: For each conformation, the electronic coupling, Γ, was calculated ab initio for the donor–acceptor pair.
4. CT rate constants: For each conformation, CT rate constants were computed using ${\left\langle U_{DA}\right\rangle }_{D}$ , ${\left\langle U_{AD}\right\rangle }_{A}$ , σ_*DA*_^2^, σ_*AD*_^2^, *C*_*UU*_(*t*), and Γ, based on the *k* ^*G*^ for the all-atom model and the hierarchy of expressions (*k* ^W–AV^ through *k* ^C–0^) for the spin-boson model.

## 3 Results

### 3.1 Representative Conformations

The inherent flexibility of the peptoid and linkers gives rise to a broad ensemble of conformations that differ with respect to the donor–acceptor geometry (see illustrated through following the Fe–Fe distance in Supporting Information Figure S1). To identify the structural DOFs most relevant to CT, principal component analysis was performed on the equilibrated trajectories. The first three principal components account for 44.8%, 18.9%, and 8.3% of the covariance, respectively, corresponding primarily to approach and separation of the TPP-containing side chains (PC1), out-of-plane TPP rotation (PC2; Θ_tilt_), and in-plane TPP rotation (PC3; Θ_*z*_), while modes 4–10 correspond to smaller-amplitude vibrational motions (see Supporting Information Figures S2 and S3). Notably, PC1 closely tracks the Fe–Fe distance and therefore provides a simple descriptor of the dominant large-amplitude conformational motion impacting CT.

To systematically investigate the associated CT dynamics, 15 conformations were selected from the equilibrated trajectories using Fe–Fe separation and relative porphyrin orientation as the primary structural descriptors as described in Section 2.2. This reduced set spans the dominant geometrical variations identified by PCA and provides a compact but physically diverse representation of the conformational ensemble for subsequent electronic-structure and rate calculations.

In addition to defining the D–A geometries used in the CT calculations, the helical scaffold generates a persistent electrostatic environment whose implications for donor–acceptor asymmetry are discussed below.

### 3.2 CT Rate Constants

In the next step, CT rate constants were calculated for the 15 representative conformations, based on the rate theory protocols outlined in Section 2.1. Within the all-atom model, LSC-based CT rate constants were calculated using $k_{j\to k}^{G}$ (eq 10). Other levels of the LSC-based hierarchy of expressions for the rate constant were employed after mapping to the spin-boson model (eqs 18–41).

The values of $\left\{{\left\langle U_{DA}\right\rangle }_{D},{\left\langle U_{AD}\right\rangle }_{A},\sigma_{DA},\sigma_{AD},\Delta E_{D\to A}\right\}$ for the 15 representative conformations are listed in Table 1. Those values were obtained by averaging over multiple trajectories generated by performing MD simulations on the all-atom model. The values of $\left\{{\left\langle U_{DA}\right\rangle }_{D},{\left\langle U_{AD}\right\rangle }_{A},\sigma_{DA},\sigma_{AD}\right\}$ vary by no more than an order of magnitude across conformations. Given that the entries in Table 1 were calculated for frozen peptoid conformations, the variation across conformations reflects variations in solvent structure and dynamics. While not insignificant, such variations are seen to be rather small, which implies that the solvent structure and dynamics are relatively insensitive to the peptoid conformation.

**Table 1:** Mean Energy Gaps ${\left\langle U_{DA}\right\rangle }_{D}$ and ${\left\langle U_{AD}\right\rangle }_{A}$ , Their Standard Deviations σ_*DA*_ and σ_*AD*_, and the Reaction Energy Δ*E*_*D*→*A*_ Are Given for the 15 Conformations (All Values in eV)

conf	${\left\langle U_{DA}\right\rangle }_{D}$	${\left\langle U_{AD}\right\rangle }_{A}$	**σ**_***DA***_	**σ**_***AD***_	**Δ** *E*_*D* **→** *A*_
1	5.60 ± 0.05 × 10^–1^	8.18 ± 0.04 × 10^–1^	1.95 ± 0.03 × 10^–1^	1.95 ± 0.03 × 10^–1^	–1.22 ± 0.10 × 10^–1^
2	5.59 ± 0.03 × 10^–1^	8.02 ± 0.06 × 10^–1^	1.88 ± 0.02 × 10^–1^	1.93 ± 0.01 × 10^–1^	–1.09 ± 0.06 × 10^–1^
3	5.59 ± 0.07 × 10^–1^	7.81 ± 0.03 × 10^–1^	1.98 ± 0.00 × 10^–1^	1.84 ± 0.02 × 10^–1^	–1.27 ± 0.06 × 10^–1^
4	5.67 ± 0.05 × 10^–1^	7.99 ± 0.02 × 10^–1^	2.08 ± 0.04 × 10^–1^	1.88 ± 0.01 × 10^–1^	–1.34 ± 0.05 × 10^–1^
5	5.73 ± 0.06 × 10^–1^	8.02 ± 0.04 × 10^–1^	2.00 ± 0.02 × 10^–1^	1.87 ± 0.02 × 10^–1^	–1.29 ± 0.09 × 10^–1^
6	5.76 ± 0.06 × 10^–1^	7.66 ± 0.04 × 10^–1^	2.02 ± 0.04 × 10^–1^	1.87 ± 0.02 × 10^–1^	–1.09 ± 0.07 × 10^–1^
7	6.21 ± 0.05 × 10^–1^	8.05 ± 0.05 × 10^–1^	2.12 ± 0.02 × 10^–1^	1.97 ± 0.03 × 10^–1^	–1.03 ± 0.07 × 10^–1^
8	6.70 ± 0.07 × 10^–1^	8.17 ± 0.04 × 10^–1^	2.12 ± 0.02 × 10^–1^	1.98 ± 0.01 × 10^–1^	–8.94 ± 0.53 × 10^–2^
9	6.89 ± 0.07 × 10^–1^	7.50 ± 0.03 × 10^–1^	2.08 ± 0.03 × 10^–1^	1.90 ± 0.03 × 10^–1^	–5.70 ± 0.60 × 10^–2^
10	8.42 ± 0.08 × 10^–1^	8.94 ± 0.04 × 10^–1^	2.20 ± 0.02 × 10^–1^	2.17 ± 0.02 × 10^–1^	–3.04 ± 0.67 × 10^–2^
11	8.03 ± 0.07 × 10^–1^	8.58 ± 0.06 × 10^–1^	2.20 ± 0.02 × 10^–1^	2.09 ± 0.01 × 10^–1^	–4.34 ± 0.51 × 10^–2^
12	7.79 ± 0.06 × 10^–1^	8.14 ± 0.07 × 10^–1^	2.05 ± 0.03 × 10^–1^	2.03 ± 0.01 × 10^–1^	–1.98 ± 0.72 × 10^–2^
13	8.27 ± 0.12 × 10^–1^	8.74 ± 0.06 × 10^–1^	2.25 ± 0.03 × 10^–1^	2.05 ± 0.02 × 10^–1^	–5.80 ± 1.20 × 10^–2^
14	6.41 ± 0.09 × 10^–1^	8.20 ± 0.04 × 10^–1^	2.16 ± 0.04 × 10^–1^	1.95 ± 0.01 × 10^–1^	–1.12 ± 0.05 × 10^–1^
15	6.00 ± 0.04 × 10^–1^	6.82 ± 0.03 × 10^–1^	1.83 ± 0.03 × 10^–1^	1.85 ± 0.02 × 10^–1^	–3.72 ± 0.67 × 10^–2^

Δ*E*_*D*→*A*_ is seen to be negative for all conformations, with values ranging between 1 – 5*k*_B_ *T*. This is consistent with our convention that the donor-to-acceptor CT reaction is downhill. The variation of Δ*E*_*D*→*A*_ across conformations indicates that the thermodynamics of the CT reaction depends on the relative orientation of the peptoid backbone with respect to the porphyrins. The moderate magnitude of Δ*E*_*D*→*A*_ also implies that the back-reaction from acceptor to donor is significant.

The asymmetry which leads to negative Δ*E*_*D*→*A*_ in all 15 conformations can be traced back to the interaction between the iron cations and the permanent dipole moment associated with the peptoid backbone, with the variation in value attributed to different relative orientations. More specifically, conformation-dependent asymmetry in Δ*E*_*D*→*A*_ has an electrostatic origin that can be quantified by considering the differential electrostatic potential, Δϕ = ϕ­(Fe_2_) – ϕ­(Fe_1_), with contributions from the peptoid scaffold, the porphyrin/heme framework, and the solvent. As illustrated in Figure 2, the helical scaffold carries a dipole of approximately 22.0 D due to its aligned *cis* amide carbonyl groups. This dipole is offset from the Fe–Fe midpoint, producing a geometry-dependent bias, Δϕ_pep_ ≈ −0.03 to +0.16 V, that contributes to the porphyrin-solvent electrostatics and therefore affecting the solvent reorganization. The persistence of this directional bias across full conformational averaging with proper free-energy weighting remains an open question beyond the scope of the present study.

**Figure 2:**
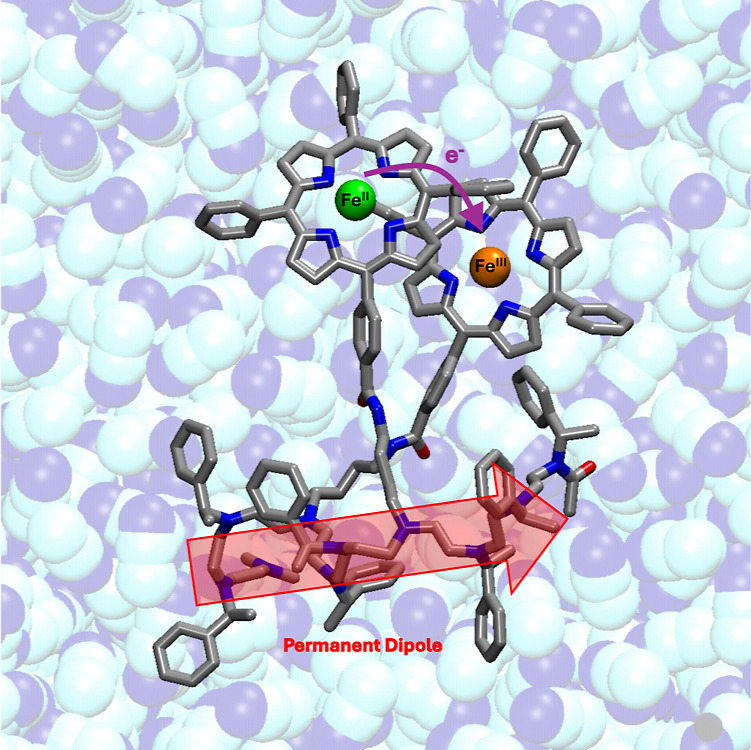
A representative peptoid–porphyrin conformation showing the scaffold dipole (red arrow) relative to the Fe–Fe axis. The dipole is offset from the Fe–Fe midpoint and asymmetrically oriented with respect to the two Fe sites, generating a nonzero differential electrostatic potential between Fe_1_ and Fe_2_.

Further insight can be obtained by considering the probability distributions of *U*_*DA*_ in thermal equilibrium on the donor and acceptor PESs (see Figure 3). The distributions are observed to be Gaussian, consistent with the assumption of Gaussian statistics. Additionally, the distributions vary across conformations, reflecting the conformation dependence of ${\left\langle U_{DA}\right\rangle }_{D}$ , ${\left\langle U_{AD}\right\rangle }_{A}$ , σ_*DA*_, and σ_*AD*_ (see Table 1). For some conformations, the widths of the Gaussian distributions differ between the donor and acceptor states, justifying our treatment of σ_*DA*_ and σ_*AD*_ as independent parameters (see eq 10).

**Figure 3:**
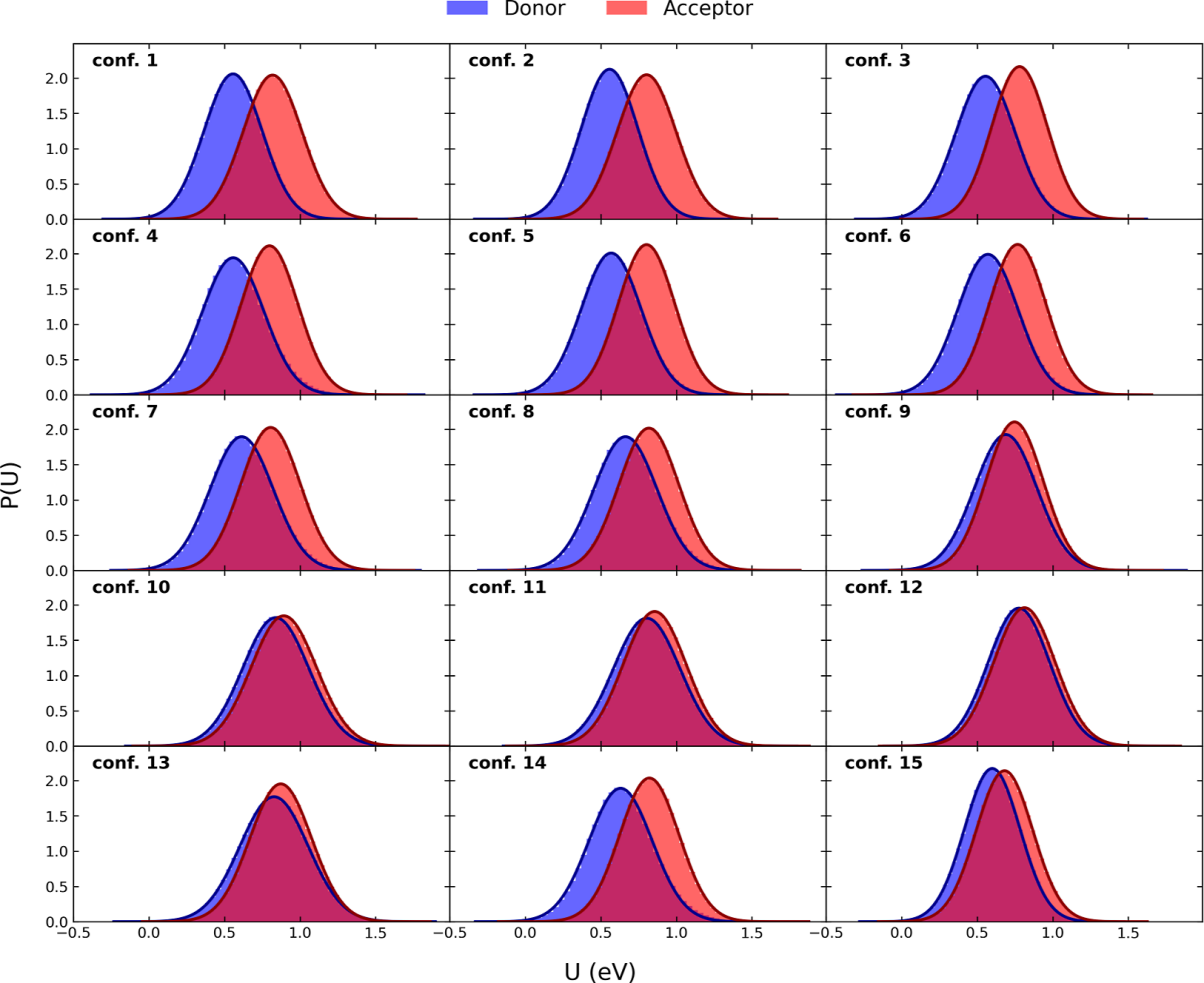
Probability distributions of *U*_*DA*_ (blue) and *U*_*AD*_ (red) in thermal equilibrium on the donor and acceptor PESs, respectively, for the 15 representative conformations. The distributions are Gaussian and vary across conformations. The corresponding averages and widths ( ${\left\langle U_{DA}\right\rangle }_{D}$ , ${\left\langle U_{AD}\right\rangle }_{A}$ , σ_*DA*_, and σ_*AD*_) are reported in Table 1. For some conformations, the widths differ between the donor and acceptor states, justifying the treatment of σ_*DA*_ and σ_*AD*_ as independent parameters.

Next, we consider the mapping of the all-atom Hamiltonian onto the spin-boson Hamiltonian. To this end, we calculated *C*_*UU*_(*t*) (eq 16) and *J*(ω) (eq 15), using the computational protocol outlined in Appendix B, for each of the 15 conformations. The results are shown in Figure 4. As expected, *C*_*UU*_(*t*) and *J*(ω) are seen to vary from one conformation to another. However, the differences are rather small. In general, *C*_*UU*_(*t*) and *J*(ω) are seen to follow similar trends in all 15 conformations, which is consistent with the fact that the solvent dynamics in the close vicinity of the peptoid is not strongly affected by its conformation.

**Figure 4:**
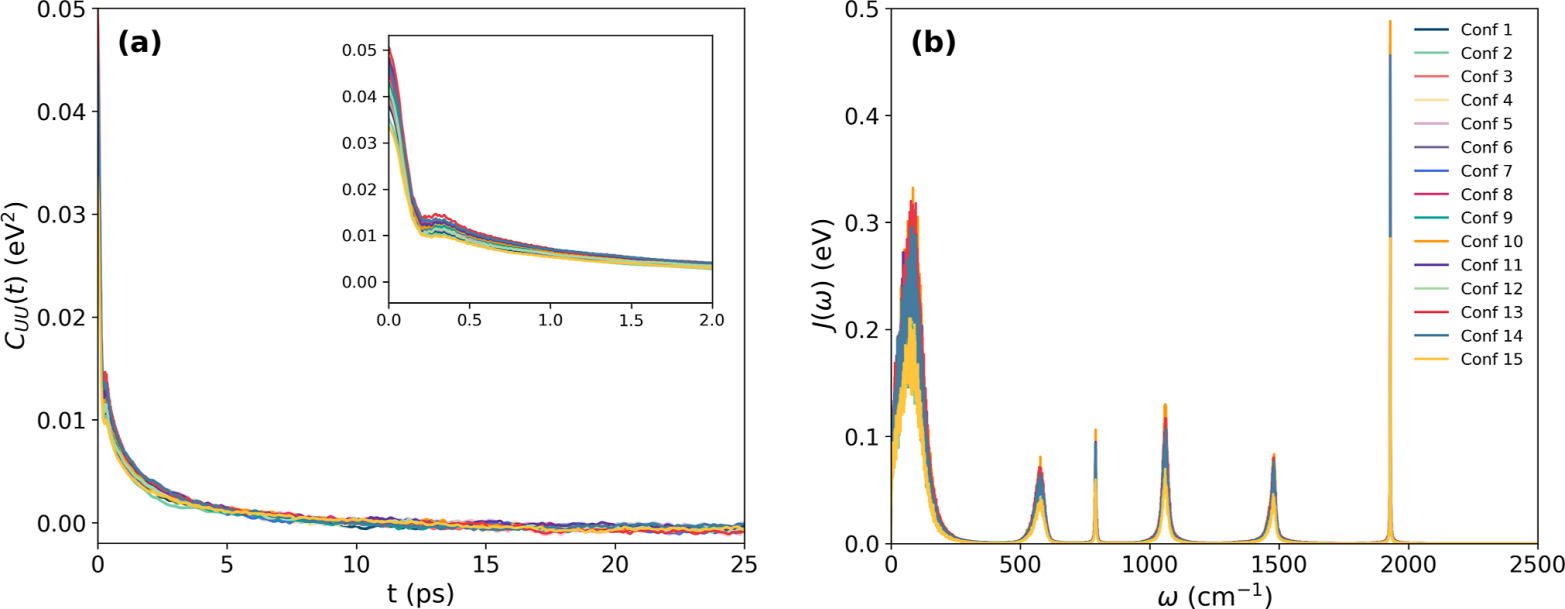
(a) Energy-gap autocorrelation functions, *C*_*UU*_(*t*) (see eq 16), and (b) Corresponding spectral densities, *J*(ω) (see eq 15), for the 15 conformations under consideration.

Once the all-atom model is mapped onto the spin-boson model, it is possible to calculate the complete hierarchy of LSC-based approximations (see eqs 18–41). The calculated *k*_*D*→*A*_/Γ^2^ across the hierarchy of approximations in eqs 18–41, listed in Table 2, are essentially the same, indicating that dynamical and quantum effects are negligible. A notable deviation is observed between $k_{D\to A}^{C-0}/\Gamma^{2}$ , obtained from the spin-boson model, and $k_{D\to A}^{G}/\Gamma^{2}$ , obtained from the all-atom model. Both rates assume Gaussian statistics, but only $k_{D\to A}^{C-0}$ enforces identical Gaussian distributions on the donor and acceptor PESs. Indeed, conformation 14, which shows the largest deviation in Gaussian statistics between donor and acceptor PESs, also shows the largest discrepancy between $k_{D\to A}^{C-0}$ and $k_{D\to A}^{G}$ .

**Table 2:** Coupling-Normalized Rate Constants, *k*_*D*→*A*_/Γ^2^, Calculated Using the Hierarchy of Approximations in eqs 18–41 ^a^

conf	$k_{D\to A}^{W-AV}$	$k_{D\to A}^{W-0}$	$k_{D\to A}^{C-AV}$	$k_{D\to A}^{C-D}$	$k_{D\to A}^{C-0}$	$k_{D\to A}^{G}$
1	2.4 × 10^0^	2.4 × 10^0^	2.3 × 10^0^	2.4 × 10^0^	2.4 × 10^0^	5.8 ± 0.7 × 10^0^
2	3.5 × 10^0^	3.5 × 10^0^	3.5 × 10^0^	3.5 × 10^0^	3.5 × 10^0^	4.3 ± 0.3 × 10^0^
3	2.2 × 10^0^	2.2 × 10^0^	2.2 × 10^0^	2.2 × 10^0^	2.2 × 10^0^	6.4 ± 0.7 × 10^0^
4	1.1 × 10^0^	1.1 × 10^0^	1.1 × 10^0^	1.1 × 10^0^	1.1 × 10^0^	8.1 ± 0.9 × 10^0^
5	2.0 × 10^0^	2.0 × 10^0^	2.0 × 10^0^	2.0 × 10^0^	2.0 × 10^0^	5.6 ± 0.5 × 10^0^
6	1.3 × 10^0^	1.3 × 10^0^	1.3 × 10^0^	1.3 × 10^0^	1.3 × 10^0^	5.7 ± 0.7 × 10^0^
7	4.8 × 10^–1^	4.8 × 10^–1^	4.7 × 10^–1^	4.7 × 10^–1^	4.7 × 10^–1^	4.5 ± 0.6 × 10^0^
8	3.7 × 10^–1^	3.8 × 10^–1^	3.7 × 10^–1^	3.7 × 10^–1^	3.7 × 10^–1^	2.2 ± 0.4 × 10^0^
9	3.2 × 10^–1^	3.2 × 10^–1^	3.2 × 10^–1^	3.2 × 10^–1^	3.2 × 10^–1^	1.3 ± 0.2 × 10^0^
10	6.4 × 10^–2^	6.5 × 10^–2^	6.4 × 10^–2^	6.4 × 10^–2^	6.4 × 10^–2^	2.1 ± 0.3 × 10^–1^
11	8.9 × 10^–2^	8.9 × 10^–2^	8.8 × 10^–2^	8.8 × 10^–2^	8.8 × 10^–2^	3.9 ± 0.6 × 10^–1^
12	2.0 × 10^–1^	2.0 × 10^–1^	2.0 × 10^–1^	2.0 × 10^–1^	2.0 × 10^–1^	2.4 ± 0.6 × 10^–1^
13	7.2 × 10^–2^	7.3 × 10^–2^	7.2 × 10^–2^	7.2 × 10^–2^	7.2 × 10^–2^	3.5 ± 1.3 × 10^–1^
14	4.2 × 10^–1^	4.2 × 10^–1^	4.2 × 10^–1^	4.2 × 10^–1^	4.2 × 10^–1^	3.9 ± 0.4 × 10^0^
15	1.4 × 10^0^	1.4 × 10^0^	1.4 × 10^0^	1.4 × 10^0^	1.4 × 10^0^	1.8 ± 0.4 × 10^0^

a The essentially identical values across the hierarchy indicate that dynamical and quantum effects are negligible.

### 3.3 Conformational Dependence of the Electronic Coupling and CT Rate Constants

The analysis thus far demonstrates that a rate theory based on E-FGR, at the level of approximation in eq 10, provides an *efficient* route for calculating *accurate* CT rate constants in peptoid-based systems under ambient conditions. Importantly, evaluating *k*_*D*→*A*_ and *k*_*A*→*D*_ using eq 10 requires five parameters: ${\left\langle U_{DA}\right\rangle }_{D}$ , ${\left\langle U_{AD}\right\rangle }_{A}$ , σ_*DA*_, σ_*AD*_, and Γ. Four of these— ${\left\langle U_{DA}\right\rangle }_{D}$ , ${\left\langle U_{AD}\right\rangle }_{A}$ , σ_*DA*_, and σ_*AD*_—were found to be relatively insensitive to the peptoid conformation. Thus, the structure and dynamics of the solvent in the close vicinity of the peptoid are largely independent of its conformation. In contrast, the electronic coupling constant, Γ, was found to be highly sensitive to the conformation, Thus, Γ emerges from our analysis as the prime target for manipulating and controlling CT rates.

Correlation analysis pointed to two key intermolecular coordinates that influence Γ: (1) the Fe–Fe distance, *R*_Fe–Fe_, and (2) the relative porphyrin tilt angle, θ_Tilt_. Specifically, both *R*_Fe–Fe_ and θ_Tilt_ emerge as intermolecular features strongly affecting Γ across the sampled conformations, with correlation coefficients of −0.47 and −0.41, respectively. The values of *R*_Fe–Fe_ and θ_Tilt_ for the 15 conformations under consideration are listed in Table 3. (Full atomic coordinates for each conformation are provided in the Supporting Information). In general, larger couplings are associated with short Fe–Fe separations and nearly parallel porphyrin planes.

**Table 3:** Electronic Couplings (Γ, meV) and Their Corresponding Intermolecular *R*_Fe–Fe_ and θ_Tilt_ Coordinates

#	Γ	*R*_Fe–Fe_	θ_Tilt_
1	0.0027	6.50	5.6
2	0.3755	5.47	11.4
3	0.4327	6.04	21.4
4	0.0435	6.29	5.0
5	3.1157	5.83	5.3
6	3.1919	5.35	14.8
7	5.0096	5.31	3.7
8	0.9932	6.32	13.0
9	0.1034	7.75	9.4
10	0.0027	8.92	52.4
11	0.0109	7.30	50.0
12	0.2259	7.11	40.8
13	0.0163	6.32	50.4
14	0.0327	5.01	8.2
15	0.3510	6.11	20.3

Across the 15 representative conformations under consideration, Γ spans nearly 3–4 orders of magnitude, reaching values as high as 5 meV, exceeding 0.4 meV for five conformations, and falling below 0.1 meV for six conformations, with a minimum value of 0.0027 meV. This wide range of Γ values is the primary origin for the conformational variability in the CT rate constants. For example, conformations 5–8, which exhibit relatively large values of Γ correspond to conformations with *R*_Fe–Fe_ < 6.5 Å and θ_Tilt_ < 15°. However, the relationship is not strictly monotonic. For example, conformation 14 correspond to a comparatively small Γ, despite the short Fe–Fe distance and small tilt angle. These observations suggest that other intramolecular coordinates can play a role in modulating Γ.

The wide range of Γ values, together with the quadratic dependence of the E-FGR CT rate constant on Γ, gives rise to a correspondingly broad distribution of donor-to-acceptor CT rate constants across the conformational ensemble. The rate constants calculated from both the all-atom model ( $k_{D\to A}^{G}$ , eq 10) and the spin-boson model ( $k_{D\to A}^{W-0}$ , eq 18) are listed in Table 4. Their conformational variation is illustrated in Figure 5a, which further demonstrates the dominant role of the electronic coupling in determining the CT rates, as evidenced by the substantially reduced variation in *k*_*D*→*A*_/Γ^2^ compared to *k*_*D*→*A*_.

**Table 4:** CT Rate Constants Calculated from the All-Atom Model ( $k_{D\to A}^{G}$ , eq 10) and the Spin-Boson Model ( $k_{D\to A}^{W-0}$ , eq 18) for Each of the 15 Conformations

conf	$k_{D\to A}^{W-AV}$ (*s* ^–1^)	$k_{D\to A}^{G}$ (*s* ^–1^)
1	9.6 × 10^2^	2.4 ± 0.3 × 10^3^
2	2.7 × 10^7^	3.4 ± 0.3 × 10^7^
3	2.3 × 10^7^	6.7 ± 0.7 × 10^7^
4	1.1 × 10^5^	8.6 ± 0.9 × 10^5^
5	1.1 × 10^9^	3.0 ± 0.2 × 10^9^
6	7.2 × 10^8^	3.2 ± 0.4 × 10^9^
7	6.7 × 10^8^	6.2 ± 0.9 × 10^9^
8	2.1 × 10^7^	1.2 ± 0.2 × 10^8^
9	5.6 × 10^5^	2.3 ± 0.3 × 10^6^
10	2.6 × 10^1^	8.4 ± 1.2 × 10^1^
11	5.9 × 10^2^	2.6 ± 0.4 × 10^3^
12	5.7 × 10^5^	6.7 ± 1.6 × 10^5^
13	1.1 × 10^3^	5.2 ± 2.0 × 10^3^
14	2.5 × 10^4^	2.3 ± 0.3 × 10^5^
15	9.6 × 10^6^	1.2 ± 0.3 × 10^7^

**Figure 5:**
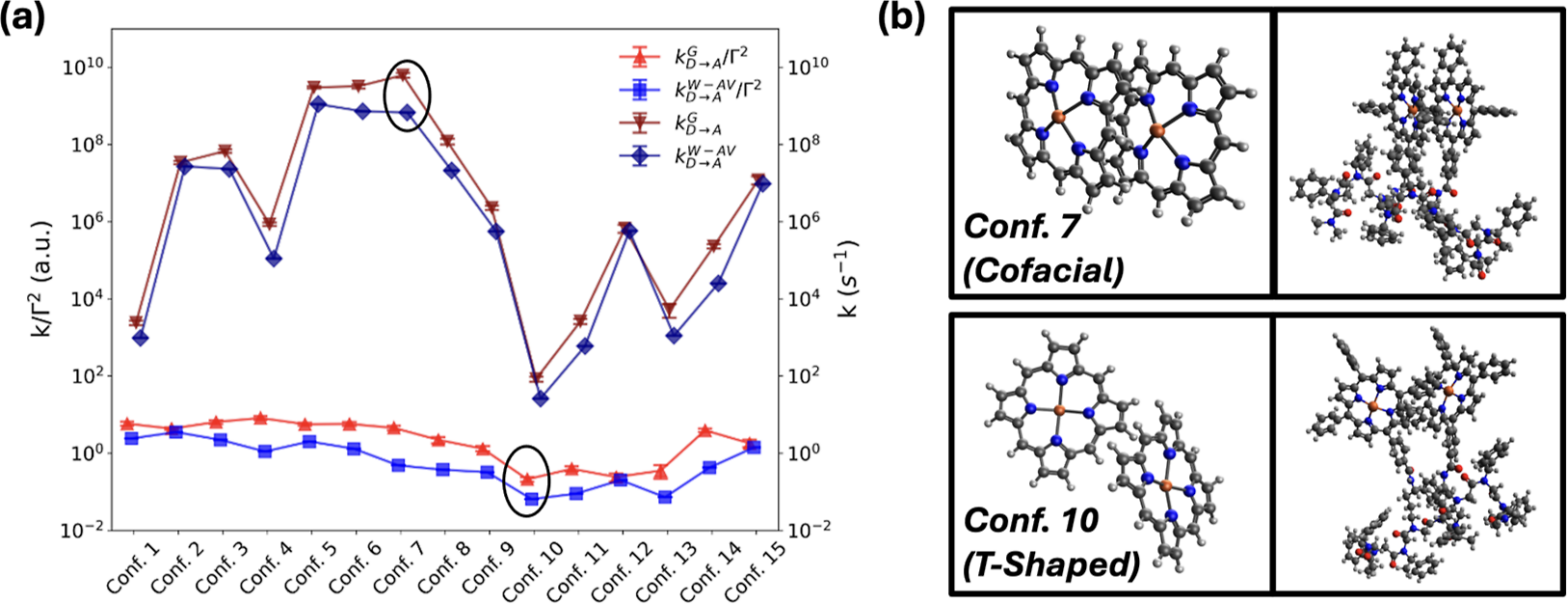
(a) Semilog plot of the conformational dependence of *k*_*D*→*A*_ and *k*_*D*→*A*_/Γ^2^. Rate constants are shown for the all-atom model ( $k_{D\to A}^{G}$ , eq 10) and the spin-boson model ( $k_{D\to A}^{W-0}$ , eq 18). The results demonstrate that the conformational variation of the CT rate is dominated by the electronic coupling, Γ. (b) Conformational snapshots corresponding to the maximum and minimum values of the CT rate constant (circled in (a)). The observed rate variation correlates strongly with the electronic coupling, Γ: The maximum-rate conformation displays a nearly π-stacked geometry, while the minimum-rate conformation is more tilted and exhibits a larger Fe–Fe distance.

Figure 5b shows representative conformations corresponding to the maximum and minimum electronic couplings (and CT rates). The maximum-coupling structure adopts a relatively well π-stacked arrangement, with a nearly vanishing tilt angle of 5*°* and a short Fe–Fe distance, whereas the minimum-coupling structure is significantly more tilted (52*°*) and exhibits a larger Fe–Fe separation of 9 Å. The corresponding orientational parameters for the full conformational ensemble are summarized in Table 3. These observations indicate that the electronic coupling is strongly influenced by intermolecular geometry. A quantitative analysis of the structural origin of the observed variations in Γ is beyond the scope of the present work and will be presented in a follow-up study.^104^

## 4 Conclusions and Discussion

The comprehensive E-FGR framework, which combines MD sampling with electronic coupling calculations, enables a quantitative connection between molecular-level structure and charge-transfer (CT) properties in soft, self-assembled materials, as demonstrated for functionalized peptoids containing iron–porphyrin units. By resolving conformational fluctuations in both the energetic landscape and the electronic coupling, this approach identifies electronic coupling as the key quantity governing CT variability across molecular conformations.

Fully capturing this conformation dependence requires a theoretical framework capable of resolving both energetic fluctuations and electronic couplings. The LSC approach, as implemented in this work and validated via mapping to the spin-boson model, provides such a framework, enabling predictive modeling of CT behavior across diverse molecular conformations.

Applying this framework, the donor-to-acceptor CT rates span nearly eight orders of magnitude across the sampled conformations. In contrast, rates normalized by setting Γ = 1 remain roughly within the same order of magnitude, indicating that the large variation in CT efficiency originates primarily from the strong conformational dependence of the electronic coupling, Γ. The coupling coefficients span three to four orders of magnitude across the sampled conformations, underscoring its dominant role in controlling CT rates in these systems. Representative conformations corresponding to the largest and smallest values of Γ are shown in Figure 5b to illustrate conformational features strongly affecting the variation in the rate.

Future work will extend this framework to further resolve the effects of conformational variability on Γ. Additional strategies for directing CT—such as local electric fields via molecular design, solvent effects, or more complex nanoscale architectures (e.g., peptoid backbones forming membranes)—will also be explored. The framework presented here provides a foundation for such studies, with the ultimate goal of enabling predictive control of charge transfer through molecular design of peptoid-based materials.

## Supplementary Material





## Appendix A

### $k_{D\to A}^{G}$ and $k_{A\to D}^{G}$ vs Marcus Theory

While the rate theory in eq 10 is based on assumptions similar to those underlying standard Marcus theory, it is not equivalent to it. In this Appendix we explain the difference between the two rate theories, and why it becomes important in the case of asymmetrical reactions with significant forward and backward rates, such as the peptoid-based system in this paper.

Importantly, calculating $k_{D\to A}^{G}$ and $k_{A\to D}^{G}$ requires four independently determined parameters: $\left\{{\left\langle U_{DA}\right\rangle }_{D},{\left\langle U_{AD}\right\rangle }_{A},\sigma_{DA}^{2},\sigma_{AD}^{2}\right\}$ . In contrast, within standard Marcus theory, the same rate constants are given in terms of only *two* parameters, namely the reorganization energy, *E*_r_, and the donor-to-acceptor reaction free energy, Δ*E*_*D*→*A*_

23
$$\begin{array}{ll} k_{D\to A}^{M} & =\frac{\Gamma^{2}}{\hbar }\sqrt{\frac{\pi }{k_{B}TE_{r}}}\exp\left[-\frac{{\left(\Delta E_{D\to A}+E_{r}\right)}^{2}}{4k_{B}TE_{r}}\right],\\ k_{A\to D}^{M} & =\frac{\Gamma^{2}}{\hbar }\sqrt{\frac{\pi }{k_{B}TE_{r}}}\exp\left[-\frac{{\left(-\Delta E_{D\to A}+E_{r}\right)}^{2}}{4k_{B}TE_{r}}\right] \end{array}$$

Consistency between $k_{D\to A}^{G}$ and $k_{D\to A}^{M}$ , dictates the following relationships between $\left\{{\left\langle U_{DA}\right\rangle }_{D},\sigma_{DA}^{2}\right\}$ and {Δ*E*_*D*→*A*_, *E*_*r*_}­

24
$$E_{r}=\frac{\sigma_{DA}^{2}}{2k_{B}T}=-\Delta E_{D\to A}-{\left\langle U_{DA}\right\rangle }_{D}$$

Similarly, consistency between the rate constants of the inverse reaction, $k_{A\to D}^{G}$ and $k_{A\to D}^{M}$ , dictate corresponding relationships, where

25
$$E_{r}=\frac{\sigma_{AD}^{2}}{2k_{B}T}=\Delta E_{D\to A}-{\left\langle U_{AD}\right\rangle }_{A}$$

Importantly, the fact that $\left\{{\left\langle U_{DA}\right\rangle }_{D},{\left\langle U_{AD}\right\rangle }_{A},\sigma_{DA}^{2},\sigma_{AD}^{2}\right\}$ are independent variables implies that eqs 24 and 25 can give rise to inconsistent values of Δ*E*_*D*→*A*_ and *E*_*r*_. We therefore refrain from using these equations to define Δ*E*_*D*→*A*_ or *E*_*r*_.

It should be noted that there are two special cases for which the rate theories in eqs 23 and 10 are equivalent:

1. A pronouncedly downhill reaction: In this case, *k*_*D*→*A*_ ≫ *k*_*A*→*D*_, so that *k*_*A*→*D*_ ∼ 0, allowing a one-to-one correspondence between $\left\{{\left\langle U_{DA}\right\rangle }_{D},\sigma_{DA}^{2}\right\}$ and {Δ*E*_*D*→*A*_, *E*_r_}.^27^
2. A symmetrical reaction: In this case, *k*_*D*→*A*_ = *k*_*A*→*D*_, implying Δ*E*_*D*→*A*_ = 0 and *σ*_*AD*_^2^ = *σ*_*DA*_^2^.

However, for an asymmetrical reaction with significant forward and backward rates, the rate theory based on eq 10 cannot be mapped onto standard Marcus theory in a self-consistent way.

This is demonstrated in Table [Table tblA1], which features the values of Δ*E*_*D*→*A*_ (in eV), as calculated for different peptoid conformations via eqs 24, 25 and 11. The fact that eqs 24 and 25 produce *different* values of Δ*E*_*D*→*A*_ demonstrates their inconsistency. We therefore refrain from using eqs 24 and 25 to define Δ*E*_*D*→*A*_ or *E*_r_. Instead, we use eq 11 to define Δ*E*_*D*→*A*_ in a way which is unambiguous and satisfies microscopic reversibility (Δ*E*_*D*→*A*_ = −Δ*E*_*A*→*D*_). As for the reorganization energy, *E*_r_, it can still be defined based on eqs 24 and 25. However, the donor’s reorganization energy, *E*_*r*_^*D*^ = *σ*_*DA*_^2^/2*k*_B_ *T*, need not be the same as the acceptor’s reorganization energy, *E*_*r*_^*A*^ = *σ*_*AD*_^2^/2*k*_B_ *T* (see Figure 3).

**A1 tblA1:** Values of Δ*E*_*D*→*A*_ (in eV) as Calculated for Different Conformations via eqs 24, 25 and 11

conf	eq 24	eq 25	eq 11
1	–0.180	–0.083	–0.122
2	–0.122	–0.082	–0.109
3	–0.200	–0.124	–0.127
4	–0.274	–0.112	–0.134
5	–0.200	–0.124	–0.129
6	–0.209	–0.087	–0.109
7	–0.251	–0.054	–0.103
8	–0.201	–0.058	–0.089
9	–0.144	–0.053	–0.057
10	–0.095	0.015	–0.030
11	–0.131	–0.012	–0.043
12	–0.030	–0.016	–0.020
13	–0.152	–0.063	–0.058
14	–0.260	–0.083	–0.112
15	–0.050	–0.018	–0.037

### B Calculating *C*_*UU*_(*t*) and *J*(ω) from MD Inputs

This section details the protocol that was employed for calculating *C*_*UU*_(*t*), eq 16, and *J*(ω), eq 15, from MD trajectories, and provides the rationale for it.

The output of the MD simulations typically consists of *N* independent trajectories, each given as a series of *U* values at *n* consecutive time steps

26
$$\begin{array}{ll} & U_{1,1},U_{1,2},...,U_{1,n}\\ & U_{2,1},U_{2,2},...,U_{2,n}\\ & \vdots \\ & U_{N,1},U_{N,2},...,U_{N,n} \end{array}$$

here, *U*_*j*,*k*_ = *U*_*j*_(*k*δ) correspond to the value of *U* at the *k*-th time step of the *j*-th trajectory, where δ is the time step, 1 ≤ *j* ≤ *N* is the trajectory index and 1 ≤ *k* ≤ *n* is the time index within the trajectory.

One way of calculating *C*_*UU*_(*t*) from the data matrix in eq 26, which we will refer to as method 1, is to calculate *C*_*UU*_(*t*) for each of the trajectories

27
$$C_{UU,j}\left(k\delta \right)=\frac{1}{n-k}\sum_{l=1}^{n-k}U_{j,l}U_{j,l+k}-{\left(\frac{1}{n}\sum_{l=1}^{n}U_{j,l}\right)}^{2}$$

followed by obtaining *C*_*UU*_(*k*δ) by averaging *C*_*UU*,*j*_(*k*δ) over the *N* trajectories

28
$$C_{UU}^{\left(1\right)}\left(k\delta \right)=\frac{1}{N}\sum_{j=1}^{N}C_{UU,j}\left(k\delta \right)$$

calculated this way, the initial value of *C*_*UU*_(*t*) is given by

29
$$C_{UU}^{\left(1\right)}\left(0\right)=\frac{1}{N}\sum_{j=1}^{N}\left[{\left\langle U^{2}\right\rangle }_{j}-{\left\langle U\right\rangle }_{j}^{2}\right]$$

where ${\left\langle U^{m}\right\rangle }_{j}$ is the average of *U* ^*m*^ along the *j*-th trajectory (*m* = 1, 2)

30
$${\left\langle U^{m}\right\rangle }_{j}=\frac{1}{n}\sum_{k=1}^{n}U_{j,k}^{m}$$

at the same time, the initial value of *C*_*UU*_(*t*), which corresponds to the variance of *U*, can be calculated by averaging over the entire data matrix, and is given by

31
$$C_{UU}\left(0\right)=\left\langle U^{2}\right\rangle -{\left\langle U\right\rangle }^{2}=\frac{1}{N}\sum_{j=1}^{N}{\left\langle U^{2}\right\rangle }_{j}-{\left(\frac{1}{N}\sum_{j=1}^{N}{\left\langle U\right\rangle }_{j}\right)}^{2}$$

importantly, *C*_*UU*_^(1)^(0)≠*C*_*UU*_(0), with the difference given by the variance of the averaged *U* across trajectories

32
$$C_{UU}\left(0\right)-C_{UU}^{\left(1\right)}\left(0\right)=\frac{1}{N}\sum_{j=1}^{N}{\left({\left\langle U\right\rangle }_{j}-\left\langle U\right\rangle \right)}^{2}$$

thus, *C*_*UU*_(0)>*C*_*UU*_^(1)^(0), such that *C*_*UU*_^(1)^(0) always underestimates *C*_*UU*_(0).

Given the central role that the initial value of *C*_*UU*_(*t*) plays in determining the CT rate constant, calculating the CT rate constant based on *C*_*UU*_^(1)^(*t*) can lead to significant discrepancies. Such discrepancies can be eliminated by changing the way we calculate *C*_*UU*_(*t*) from the data matrix as follows

33
$$\begin{array}{lll} C_{UU}^{\left(2\right)}\left(k\delta \right) & = & \frac{1}{N}\sum_{j=1}^{N}\left[\frac{1}{n-k}\sum_{l=1}^{n-k}U_{j,l}U_{j,l+k}\right]\\ & & -{\left(\frac{1}{N}\sum_{j=1}^{N}{\left\langle U\right\rangle }_{j}\right)}^{2} \end{array}$$

It should be noted that *C*_*UU*_^(2)^(0) = *C*_*UU*_(0). We refer to calculating *C*_*UU*_(*t*) based on eq 33 as method 2.

Unfortunately, while method 2 removes the discrepancy at the initial time, it does so at the expense of adding a discrepancy at long times. Specifically

34
$$C_{UU}^{\left(2\right)}\left(k\delta \right)\overset{k\gg 1}{\to }\frac{1}{N}\sum_{j=1}^{N}\langle {U\rangle }_{j}^{2}-\langle {U\rangle }^{2}\geq 0$$

the fact that *C*_*UU*_^(2)^(*k*δ) does not vanish at long times, *k* ≫ 1, is clearly nonphysical. To remove the long time discrepancy without bringing back the short time discrepancy, we renormalized the correlation function as follows

35
$$C_{UU}^{\left(3\right)}\left(k\delta \right)=\frac{1}{N}\sum_{j=1}^{N}\frac{\sigma^{2}}{\sigma_{j}^{2}}C_{UU,j}\left(k\delta \right)$$

where σ^2^ = *C*_*UU*_(0) = ⟨*U* ^2^⟩ – ⟨*U*⟩^2^ and $\sigma_{j}^{2}=C_{UU,j}\left(0\right)={\left\langle U^{2}\right\rangle }_{j}-{\left\langle U\right\rangle }_{j}^{2}$ . We refer to calculating *C*_*UU*_(*t*) based on eq 35 as method 3. Importantly, *C*_*UU*_^(3)^(*k*δ) is well behaved at both short and long time, since it coincides with *C*_*UU*_(0) at the initial time and vanishes at long times. The results reported in this paper were based on correlation functions calculated via method 3.

The aforementioned discrepancy associated with method 1 is demonstrated in Table [Table tblA2], which shows the values of the reorganization energy on the donor PES, *E*_*r*_ = *C*_*UU*_(0)/(2*k*_B_ *T*), for the 15 peptoid conformations under consideration, as calculated based on methods 1,2 and 3 (note that methods 2 and 3 predict the same *C*_*UU*_(0) by design, and therefore the same value of *E*_*r*_). Method 1 is seen to underestimate *E*_r_ by 16–25% across conformations, which corresponds to a significant discrepancy.

**A2 tblA2:** Comparison of Reorganization Energies, *E*_r_ = *C*_*UU*_(0)/(2*k*_*B*_ *T*), in eV, for the 15 Peptoid Conformations Under Consideration, as Calculated Based on Methods 1,2 and 3 (Note That Methods 2 and 3 Predict the Same *C*_*UU*_(0) by Design, and Therefore the Same Value of *E*_r_)

conf	method 1	method 2/3	relative error (%)
1	0.550	0.735	–25.2
2	0.550	0.719	–23.5
3	0.550	0.657	–16.3
4	0.570	0.686	–16.9
5	0.550	0.678	–18.9
6	0.540	0.679	–20.5
7	0.570	0.750	–24.0
8	0.600	0.760	–21.1
9	0.550	0.697	–21.1
10	0.700	0.909	–23.0
11	0.660	0.846	–22.0
12	0.640	0.798	–19.8
13	0.640	0.810	–21.0
14	0.582	0.738	–21.1
15	0.520	0.664	–21.7

Finally, we note that *C*_*UU*_^(1)^(*t*) and *C*_*UU*_^(2)^(*t*) differ by a constant shift

36
$$C_{UU}^{\left(2\right)}\left(t\right)-C_{UU}^{\left(1\right)}\left(t\right)=\frac{1}{N}\sum_{j=1}^{N}{\left({\left\langle U\right\rangle }_{j}-\left\langle U\right\rangle \right)}^{2}$$

as a result, the corresponding spectral densities calculated based on *C*_*UU*_^(1)^(*t*) and *C*_*UU*_^(2)^(*t*) are identical (see eq 15)­

37
$$\begin{array}{lll} J^{\left(2\right)}\left(\omega \right)-J^{\left(1\right)}\left(\omega \right) & = & \frac{1}{N}\sum_{j=1}^{N}{\left(\langle {U\rangle }_{j}-\left\langle U\right\rangle \right)}^{2}\beta \omega \int_{0}^{\infty }dt\cos\left(\omega t\right)\\ & = & \frac{1}{N}\sum_{j=1}^{N}{\left(\langle {U\rangle }_{j}-\left\langle U\right\rangle \right)}^{2}\pi \beta \omega \delta \left(\omega \right)=0 \end{array}$$

Thus, the discrepancies between methods 1 and 2 in the time domain has no impact on the frequency domain spectral densities extracted from them.

### C Closed Form Expressions for the Approximate Expressions *C*_*D*→*A*_(*t*) in the Spin-Boson Representation

Within the spin-boson framework, eq 18 can be further simplified to the following hierarchy of closed form approximate expressions for *C*_*D*→*A*_(*t*) described in Section 2.1.2

38
$$C_{D\to A}^{W‐0}\left(t\right)=\Gamma^{2}\;\exp\left\{-\frac{i}{\hbar }\Delta Et-\sum_{j=1}^{N}\frac{\omega_{j}{\left(R_{j}^{eq}\right)}^{2}}{2\hbar }\times \left[\coth\left(\frac{\beta \hbar \omega_{j}}{2}\right)\frac{\omega_{j}^{2}t^{2}}{2}+i\omega_{j}t\right]\right\}$$

39
$$C_{D\to A}^{C‐AV}\left(t\right)=\Gamma^{2}\exp\left\{-\frac{i}{\hbar }\Delta Et-\sum_{j=1}^{N}\frac{\omega_{j}{\left(R_{j}^{eq}\right)}^{2}}{2\hbar }\times \left[\frac{2}{\beta \hbar \omega_{j}}\left(1-\cos\left(\omega_{j}t\right)\right)+i\;\sin\left(\omega_{j}t\right)\right]\right\}$$

40
$$C_{D\to A}^{C‐D}\left(t\right)=\Gamma^{2}\exp\left\{-\frac{i}{\hbar }\Delta Et-\sum_{j=1}^{N}\frac{\omega_{j}{\left(R_{j}^{eq}\right)}^{2}}{2\hbar }\times \left[\frac{2}{\beta \hbar \omega_{j}}\left(1-\cos\left(\omega_{j}t\right)\right)+i\omega_{j}t\right]\right\}$$

41
$$C_{D\to A}^{C‐0}\left(t\right)=\Gamma^{2}\exp\left\{-\frac{i}{\hbar }\Delta Et-\sum_{j=1}^{N}\frac{\omega_{j}{\left(R_{j}^{eq}\right)}^{2}}{2\hbar }\times \left[\frac{\omega_{j}t^{2}}{\beta \hbar }+i\omega_{j}t\right]\right\}$$

Unlike for the all-atom model, the fact that the entire hierarchy of approximations is given in closed form in the case of the spin-boson model, makes their calculation feasible.

## Notes

Figures derived from the molecular dynamics trajectories, including Fe–Fe distance fluctuations and principal component analyses (normalized distributions and time-dependent projections); atomic coordinates of the 15 selected dimer configurations; and a compressed archive (PDF)
ZIP file containing text files listing the atomic coordinates of all dimer configurations. (ZIP)
