# Supplementary material for: Charge Transport in Solvated Donor–Acceptor Functionalized Peptoids: Molecular Dynamics and Rate Theory
Source: J Chem Theory Comput. 2026 Jul 23;22(15):7897–912. doi: 10.1021/acs.jctc.6c00787 (PMC13472126; doi:10.1021/acs.jctc.6c00787)
Supplement: Supplementary file 1 [file ct6c00787_si_001.pdf]

Supporting Information: Charge Transport in Solvated Donor-Acceptor Functionalized Peptoids: Molecular Dynamics and Rate Theory

Zongwei Huang,[†,§] Bradley S Harris,[‡,§] Slater T. Bakenhaster,[¶] Roshan Khatri,[¶] Margaret S. Cheung,[‡] Barry D. Dunietz,[*,¶] Marcel D. Baer,[*,‡] and Eitan Geva[*,†]

†*Department of Chemistry, University of Michigan, Ann Arbor, Michigan 48109, United States*

‡*Physical Sciences Division, Pacific Northwest National Laboratory, Richland, Washington 99352, United States*

¶*Department of Chemistry and Biochemistry, Kent State University, Kent, Ohio 44242, United States*

§*Contributed equally to this work*

Received June 20, 2026; E-mail: bdunietz@ksu.edu; marcel.baer@pnnl.gov; eitan@umich.edu

SI Figures

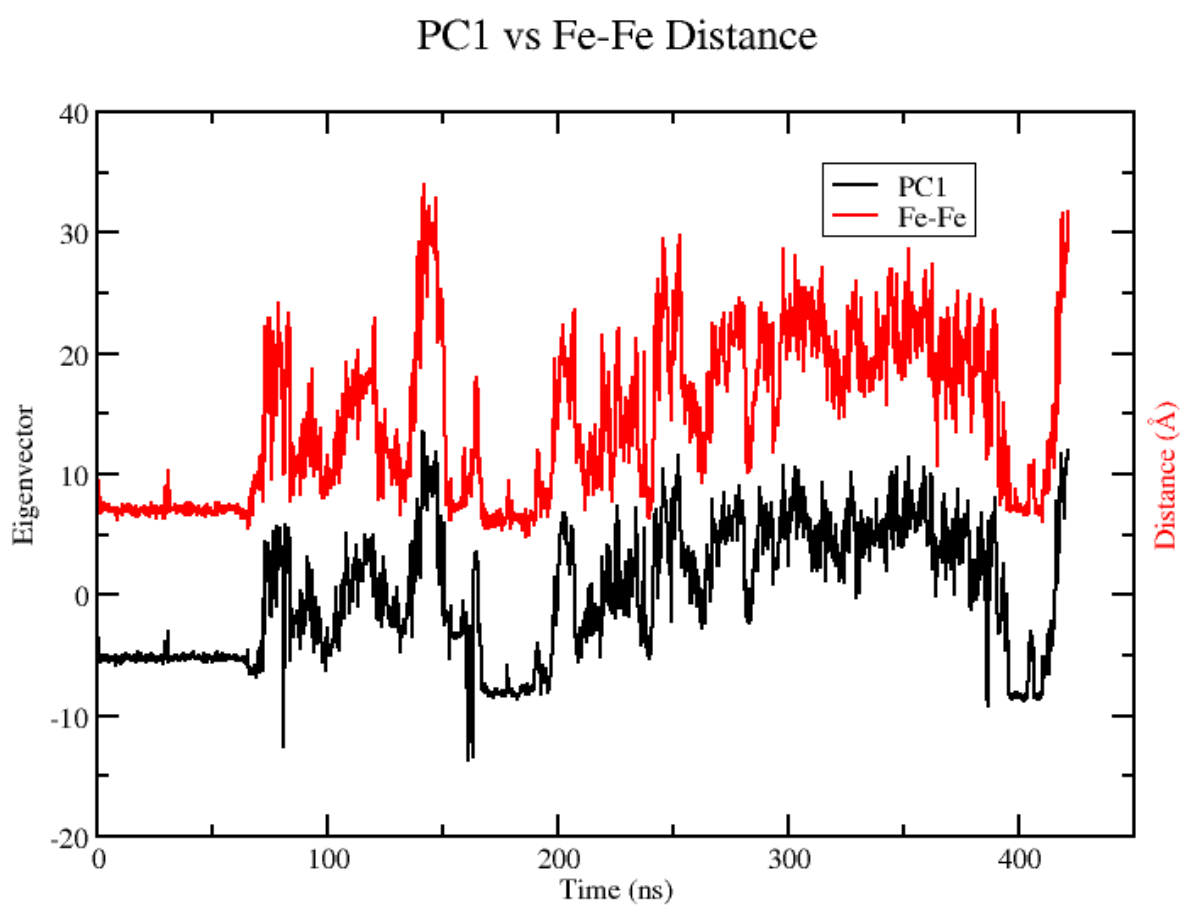

Figure S1. Variation of Iron–Iron distance (red) and principal component 1 [PC1] (black) over simulation time

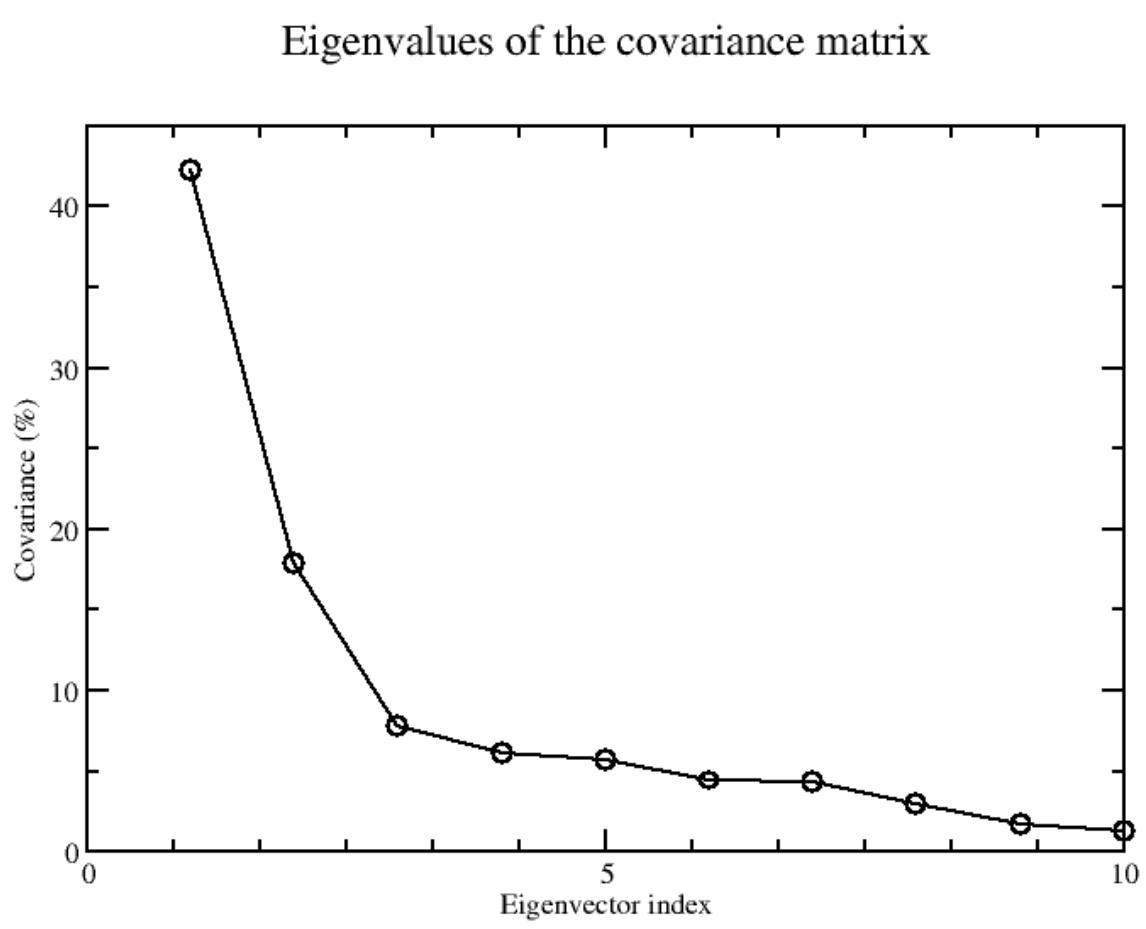

Figure S2. Principal component analysis covariance normalized to percentage

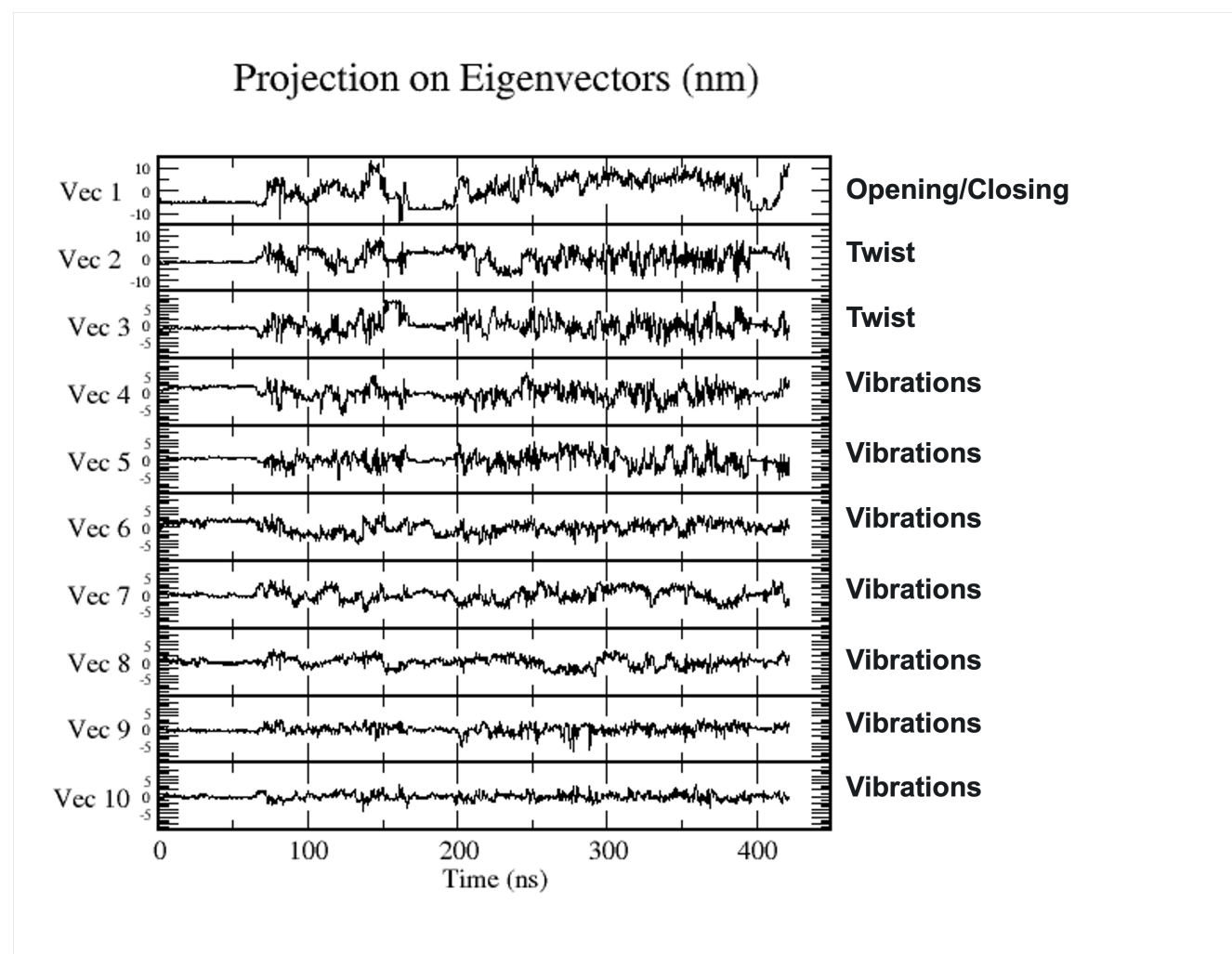

Figure S3. Principal component analysis eigenvector projections vs simulation time

Conformations

The Cartesian (XYZ) coordinates are listed next and are additionally provided in the attached SI text file for convenience.

Table S1. Structure 1 XYZ Coordinates

Atom	X	Y	Z
C	27.330	29.730	28.750
C	26.100	29.140	29.180
C	25.240	29.660	30.130
C	24.160	28.790	30.190
C	24.500	27.730	29.390
C	23.640	26.580	29.000
C	24.060	25.350	28.460
N	25.340	25.140	27.990
C	25.230	23.890	27.530
C	26.390	23.250	26.850
C	27.530	23.890	26.400
C	28.550	23.180	25.690
C	29.430	24.160	25.310
C	28.930	25.380	25.830
N	27.830	25.230	26.530
C	29.530	26.580	25.760
C	29.350	27.710	26.650
C	30.240	28.750	26.930
C	29.510	29.640	27.570
C	28.290	29.130	27.970
N	28.120	27.910	27.330
C	23.910	23.340	27.700
C	23.170	24.230	28.380
N	25.590	27.980	28.670
H	25.460	30.320	30.960
H	23.620	28.670	31.110
H	28.460	22.210	25.210
H	30.340	23.870	24.790
H	31.260	28.820	26.560
H	29.540	30.670	27.220
H	23.530	22.360	27.410
H	22.120	24.140	28.660
Fe	26.500	26.760	27.280
H	27.514	30.699	29.054
H	22.624	26.697	29.141
H	26.338	22.230	26.700
H	30.196	26.713	24.983
C	34.270	27.070	27.630
C	32.990	27.020	28.390
C	32.500	27.900	29.260
C	31.300	27.440	29.690
C	31.200	26.160	29.240
C	30.240	25.120	29.610
C	30.070	23.850	29.210
N	30.950	23.240	28.350
C	30.530	21.930	28.390
C	31.150	20.920	27.580
C	32.350	20.980	26.950
C	32.930	19.950	26.180
C	33.970	20.560	25.550
C	34.180	21.820	26.230
N	33.160	22.150	26.990
C	35.160	22.710	25.790
C	35.380	24.090	26.240
C	36.500	24.850	26.070
C	36.110	26.120	26.400
C	34.810	26.060	26.920
N	34.310	24.770	26.810
C	29.420	21.840	29.260
C	29.070	23.020	29.710
N	32.150	25.940	28.320
H	32.910	28.880	29.480
H	30.620	27.990	30.320
H	32.250	19.230	25.720
H	34.590	20.180	24.740
H	37.400	24.640	25.520
H	36.280	26.890	25.650
H	28.850	20.920	29.320
H	28.330	23.160	30.500
Fe	32.420	24.080	27.270
H	34.791	27.960	27.649
H	29.556	25.424	30.321
H	30.619	20.043	27.463
H	35.804	22.358	25.065

Table S2. Structure 2 XYZ Coordinates

Atom	X	Y	Z
C	28.056	29.662	27.291
C	26.721	29.240	27.762
C	25.970	29.914	28.647
C	24.765	29.164	28.810
C	24.970	28.022	28.077
C	24.049	26.905	27.947
C	24.276	25.624	27.506
N	25.465	25.260	26.977
C	25.482	23.965	26.899
C	26.644	23.125	26.594
C	27.843	23.529	26.055
C	28.862	22.577	25.665
C	29.768	23.450	25.143
C	29.318	24.783	25.250
N	28.132	24.855	25.861
C	29.959	25.929	24.841
C	29.892	27.280	25.421
C	30.828	28.336	25.468
C	30.257	29.341	26.179
C	28.992	28.940	26.550
N	28.751	27.580	26.117
C	24.129	23.483	27.238
C	23.343	24.572	27.488
N	26.145	28.073	27.409
H	26.155	30.813	29.212
H	24.056	29.421	29.590
H	28.864	21.491	25.639
H	30.693	23.186	24.651
H	31.799	28.390	24.988
H	30.770	29.969	26.899
H	23.809	22.452	27.240
H	22.316	24.596	27.830
Fe	26.922	26.598	26.131
H	28.335	30.623	27.545
H	23.077	27.105	28.229
H	26.552	22.119	26.807
H	30.570	25.828	24.015
C	33.301	26.983	27.939
C	32.098	26.806	28.800
C	31.649	27.735	29.716
C	30.337	27.324	30.112
C	30.292	26.061	29.558
C	29.325	25.061	30.003
C	29.246	23.769	29.618
N	30.133	23.114	28.811
C	29.884	21.839	28.913
C	30.558	20.767	28.183
C	31.685	20.948	27.378
C	32.289	19.847	26.759
C	33.258	20.355	26.052
C	33.232	21.801	26.352
N	32.274	22.112	27.194
C	34.203	22.710	25.910
C	34.383	24.147	26.266
C	35.476	25.039	25.939
C	34.973	26.260	26.289
C	33.831	26.048	27.019
N	33.371	24.741	26.991
C	28.696	21.708	29.736
C	28.265	22.867	30.191
N	31.302	25.736	28.735
H	31.992	28.763	29.753
H	29.529	27.907	30.518
H	31.995	18.812	26.942
H	33.783	19.786	25.291
H	36.263	24.769	25.246
H	35.118	26.943	25.459
H	28.257	20.734	29.912
H	27.624	23.097	31.040
Fe	31.526	23.993	27.529
H	33.800	27.882	28.023
H	28.622	25.378	30.689
H	30.176	19.812	28.268
H	34.892	22.330	25.242

Table S3. Structure 3 XYZ Coordinates

Atom	X	Y	Z
C	28.783	28.315	29.521
C	28.154	28.230	30.844
C	28.366	29.091	31.917
C	27.656	28.509	32.962
C	27.322	27.244	32.489
C	27.014	26.093	33.306
C	26.884	24.757	33.028
N	27.041	24.321	31.740
C	26.897	22.983	31.860
C	26.793	22.085	30.701
C	26.807	22.531	29.381
C	26.507	21.652	28.315
C	26.431	22.405	27.222
C	26.797	23.739	27.667
N	26.989	23.781	28.973
C	26.814	24.867	26.853
C	27.544	26.147	27.077
C	28.425	26.879	26.228
C	29.067	27.712	27.132
C	28.582	27.501	28.419
N	27.633	26.561	28.422
C	26.877	22.591	33.222
C	26.787	23.708	33.992
N	27.489	27.135	31.198
H	28.939	30.012	31.972
H	27.257	29.072	33.790
H	26.570	20.562	28.338
H	26.177	22.058	26.221
H	28.707	26.584	25.225
H	29.713	28.494	26.749
H	26.869	21.575	33.594
H	26.692	23.801	35.067
Fe	26.922	25.515	30.003
H	29.463	29.081	29.396
H	26.861	26.323	34.300
H	26.705	21.071	30.874
H	26.253	24.811	25.989
C	32.847	27.398	26.260
C	32.461	26.408	27.278
C	33.010	26.327	28.575
C	32.436	25.206	29.111
C	31.648	24.711	28.135
C	30.912	23.439	28.204
C	30.248	22.737	27.243
N	30.099	23.148	25.929
C	29.591	22.078	25.321
C	29.478	22.059	23.865
C	29.761	23.053	23.005
C	29.459	22.863	21.625
C	29.938	23.997	21.143
C	30.390	24.837	22.205
N	30.296	24.215	23.402
C	30.935	26.091	21.992
C	31.720	26.819	22.973
C	32.609	27.966	22.760
C	32.777	28.378	24.038
C	32.469	27.381	24.964
N	31.640	26.475	24.322
C	29.362	20.996	26.293
C	29.694	21.433	27.507
N	31.581	25.449	27.020
H	33.622	27.065	29.087
H	32.431	24.934	30.155
H	29.102	22.009	21.066
H	29.899	24.296	20.099
H	32.816	28.422	21.801
H	32.556	29.412	24.259
H	29.008	19.982	26.172
H	29.716	20.833	28.405
Fe	30.526	25.061	25.177
H	33.459	28.172	26.563
H	30.896	23.004	29.140
H	29.141	21.178	23.447
H	30.773	26.538	21.076

Table S4. Structure 4 XYZ Coordinates

Atom	X	Y	Z
C	28.538	29.043	30.433
C	27.614	28.501	31.452
C	27.749	28.623	32.828
C	26.604	28.012	33.365
C	26.028	27.317	32.347
C	24.940	26.409	32.502
C	24.450	25.638	31.504
N	24.901	25.747	30.180
C	24.333	24.663	29.641
C	24.519	24.294	28.254
C	25.198	25.004	27.280
C	25.302	24.582	25.958
C	26.032	25.563	25.318
C	26.197	26.618	26.362
N	25.780	26.203	27.521
C	26.920	27.790	26.188
C	27.802	28.393	27.135
C	28.892	29.211	26.911
C	29.420	29.312	28.165
C	28.512	28.838	29.079
N	27.531	28.154	28.437
C	23.529	23.889	30.586
C	23.562	24.579	31.723
N	26.539	27.715	31.124
H	28.206	29.464	33.334
H	26.154	28.240	34.325
H	24.995	23.606	25.584
H	26.350	25.632	24.290
H	29.256	29.707	26.017
H	30.466	29.056	28.203
H	23.153	22.884	30.434
H	23.169	24.359	32.712
Fe	25.781	27.345	29.219
H	29.295	29.650	30.785
H	24.499	26.335	33.432
H	24.097	23.400	27.958
H	26.809	28.274	25.283
C	33.028	27.678	28.325
C	31.998	26.800	28.880
C	31.853	26.563	30.169
C	30.653	25.734	30.187
C	30.365	25.412	28.909
C	29.429	24.353	28.423
C	29.090	24.036	27.106
N	29.705	24.641	26.008
C	29.194	23.958	24.916
C	29.470	24.300	23.529
C	30.423	25.171	23.054
C	30.626	25.499	21.725
C	31.658	26.394	21.695
C	32.017	26.571	23.071
N	31.322	25.776	23.889
C	32.988	27.431	23.505
C	33.374	27.727	24.861
C	34.542	28.513	25.210
C	34.354	28.725	26.577
C	33.344	27.946	27.012
N	32.628	27.333	25.962
C	28.258	22.959	25.422
C	28.166	23.045	26.747
N	31.181	26.089	28.094
H	32.542	26.539	31.010
H	30.359	25.277	31.128
H	30.146	25.179	20.809
H	31.913	27.027	20.842
H	35.113	29.139	24.527
H	34.587	29.645	27.095
H	27.838	22.149	24.826
H	27.445	22.594	27.418
Fe	31.055	26.223	25.986
H	33.605	28.171	29.024
H	28.975	23.783	29.154
H	28.883	23.835	22.819
H	33.510	27.931	22.768

Table S5. Structure 5 XYZ Coordinates

Atom	X	Y	Z
C	28.318	28.970	31.257
C	27.455	28.115	32.102
C	27.553	28.024	33.475
C	26.642	27.072	33.834
C	26.076	26.663	32.691
C	24.962	25.677	32.576
C	24.408	25.132	31.438
N	24.818	25.433	30.150
C	24.125	24.618	29.369
C	24.241	24.601	27.953
C	25.083	25.370	27.209
C	25.013	25.388	25.812
C	25.830	26.332	25.454
C	26.320	26.994	26.664
N	25.923	26.361	27.779
C	27.074	28.118	26.661
C	27.813	28.685	27.788
C	28.831	29.694	27.780
C	29.267	29.705	29.043
C	28.408	28.941	29.866
N	27.534	28.214	29.025
C	23.238	23.818	30.193
C	23.414	24.085	31.535
N	26.508	27.275	31.624
H	28.380	28.197	34.147
H	26.819	26.371	34.637
H	24.410	24.824	25.115
H	25.933	26.644	24.422
H	29.290	30.303	26.999
H	30.319	29.453	29.095
H	22.621	22.996	29.845
H	22.971	23.646	32.427
Fe	25.882	27.139	29.633
H	28.913	29.657	31.746
H	24.551	25.361	33.468
H	23.631	23.945	27.441
H	27.134	28.630	25.767
C	32.824	28.120	28.703
C	31.800	27.091	29.138
C	31.695	26.631	30.433
C	30.639	25.670	30.348
C	30.286	25.632	29.057
C	29.401	24.541	28.482
C	29.158	24.354	27.134
N	29.552	25.263	26.146
C	29.074	24.723	25.022
C	29.392	25.135	23.705
C	30.307	26.046	23.334
C	30.542	26.383	21.955
C	31.466	27.466	22.088
C	31.789	27.563	23.445
N	31.096	26.764	24.257
C	32.498	28.644	23.941
C	33.053	28.867	25.305
C	34.003	29.812	25.815
C	34.138	29.457	27.115
C	33.178	28.568	27.465
N	32.489	28.118	26.338
C	28.381	23.506	25.275
C	28.417	23.260	26.582
N	31.009	26.504	28.300
H	32.222	26.923	31.335
H	30.248	25.151	31.217
H	30.131	26.036	21.010
H	31.926	28.114	21.363
H	34.551	30.578	25.280
H	35.096	29.037	27.412
H	27.933	22.868	24.525
H	28.210	22.312	27.065
Fe	30.686	27.010	26.326
H	33.344	28.558	29.479
H	28.948	23.892	29.145
H	28.861	24.684	22.944
H	32.667	29.407	23.267

Table S6. Structure 6 XYZ Coordinates

Atom	X	Y	Z
C	28.826	28.655	31.335
C	28.091	27.721	32.199
C	28.322	27.616	33.503
C	27.505	26.699	34.010
C	26.709	26.348	32.918
C	25.571	25.330	32.905
C	25.041	24.805	31.790
N	25.401	25.159	30.467
C	24.695	24.304	29.682
C	24.671	24.390	28.209
C	25.247	25.385	27.465
C	24.927	25.475	26.094
C	25.404	26.726	25.846
C	26.075	27.195	26.975
N	26.022	26.354	28.011
C	26.717	28.416	27.059
C	27.683	28.858	28.092
C	28.860	29.688	27.925
C	29.393	29.698	29.161
C	28.662	28.863	29.988
N	27.686	28.212	29.269
C	23.907	23.453	30.473
C	24.252	23.624	31.764
N	27.084	26.990	31.805
H	28.855	28.327	34.121
H	27.282	26.584	35.072
H	24.050	24.981	25.701
H	25.241	27.345	24.975
H	29.166	30.170	27.009
H	30.463	29.826	29.233
H	23.154	22.759	30.123
H	23.848	23.045	32.585
Fe	26.185	26.914	30.025
H	29.553	29.223	31.797
H	25.185	25.026	33.812
H	24.180	23.639	27.698
H	26.498	29.099	26.317
C	32.823	28.605	28.613
C	32.080	27.410	29.013
C	32.272	26.751	30.225
C	31.430	25.646	30.161
C	30.710	25.828	28.965
C	29.706	24.889	28.425
C	29.077	24.878	27.207
N	29.449	25.753	26.246
C	28.822	25.280	25.140
C	28.810	25.957	23.856
C	29.442	27.155	23.509
C	29.358	27.795	22.216
C	30.012	28.949	22.429
C	30.530	28.986	23.763
N	30.143	27.929	24.471
C	31.401	29.938	24.220
C	32.232	29.855	25.440
C	33.398	30.543	25.798
C	33.427	30.425	27.145
C	32.768	29.299	27.441
N	31.773	29.077	26.488
C	28.189	24.112	25.473
C	28.324	23.793	26.798
N	31.105	26.865	28.264
H	33.067	26.868	30.946
H	31.688	24.743	30.692
H	29.000	27.339	21.297
H	30.076	29.694	21.649
H	34.023	31.148	25.146
H	33.370	31.336	27.724
H	27.750	23.363	24.817
H	28.074	22.912	27.382
Fe	30.204	27.601	26.559
H	33.481	28.974	29.317
H	29.437	24.124	29.063
H	28.268	25.498	23.107
H	31.486	30.799	23.657

Table S7. Structure 7 XYZ Coordinates

Atom	X	Y	Z
C	27.480	29.845	28.242
C	26.589	29.470	29.385
C	25.965	30.397	30.262
C	25.217	29.568	31.150
C	25.652	28.326	30.858
C	25.312	27.090	31.609
C	25.714	25.793	31.406
N	26.662	25.470	30.443
C	26.776	24.140	30.567
C	27.679	23.229	29.788
C	28.383	23.608	28.682
C	29.286	22.791	27.971
C	29.663	23.490	26.869
C	29.033	24.803	26.983
N	28.227	24.855	28.026
C	29.153	25.875	26.126
C	28.823	27.280	26.337
C	29.440	28.373	25.700
C	28.947	29.478	26.303
C	28.115	29.033	27.348
N	28.066	27.672	27.437
C	25.942	23.666	31.602
C	25.177	24.665	32.027
N	26.407	28.226	29.770
H	26.275	31.422	30.415
H	24.871	29.982	32.097
H	29.310	21.711	28.080
H	30.396	23.225	26.117
H	30.197	28.266	24.933
H	28.658	30.294	25.660
H	25.835	22.636	31.948
H	24.402	24.675	32.783
Fe	26.974	26.534	28.795
H	27.635	30.857	28.112
H	24.674	27.228	32.408
H	27.774	22.253	30.109
H	29.534	25.650	25.194
C	32.646	29.500	26.748
C	31.954	29.327	28.024
C	31.423	30.303	28.837
C	31.052	29.677	30.027
C	31.331	28.368	29.773
C	31.160	27.356	30.810
C	31.415	25.986	30.768
N	31.936	25.381	29.692
C	32.149	24.180	30.066
C	32.785	23.158	29.166
C	33.375	23.355	27.964
C	34.204	22.318	27.404
C	34.722	22.935	26.309
C	34.281	24.310	26.301
N	33.442	24.582	27.322
C	34.672	25.326	25.420
C	34.238	26.761	25.385
C	34.713	27.737	24.546
C	33.877	28.788	24.859
C	33.257	28.577	26.011
N	33.325	27.229	26.350
C	31.728	23.914	31.412
C	31.249	25.084	31.888
N	31.814	28.107	28.564
H	31.106	31.304	28.549
H	30.534	30.181	30.842
H	34.652	21.453	27.886
H	35.517	22.582	25.663
H	35.483	27.776	23.779
H	33.332	29.295	24.074
H	31.739	22.971	31.941
H	31.197	25.279	32.950
Fe	32.176	26.248	27.768
H	32.662	30.461	26.371
H	30.796	27.707	31.710
H	32.770	22.184	29.507
H	35.352	25.049	24.695

Table S8. Structure 8 XYZ Coordinates

Atom	X	Y	Z
C	26.358	29.429	29.687
C	25.544	28.478	30.425
C	24.776	28.848	31.496
C	24.430	27.586	32.124
C	24.866	26.644	31.223
C	24.793	25.212	31.472
C	25.457	24.244	30.772
N	26.272	24.407	29.703
C	26.826	23.203	29.524
C	27.979	22.988	28.650
C	28.539	23.934	27.814
C	29.630	23.611	26.991
C	29.889	24.778	26.332
C	28.899	25.724	26.829
N	28.158	25.261	27.868
C	28.757	27.026	26.372
C	28.328	28.159	27.124
C	28.581	29.507	26.909
C	28.073	30.109	27.991
C	27.281	29.189	28.677
N	27.534	27.900	28.189
C	26.122	22.211	30.334
C	25.333	22.865	31.163
N	25.529	27.211	30.200
H	24.463	29.811	31.887
H	23.667	27.478	32.891
H	30.205	22.687	26.962
H	30.403	24.933	25.388
H	28.976	29.884	25.971
H	28.784	30.542	28.675
H	26.503	21.195	30.395
H	24.774	22.438	31.978
Fe	26.539	26.095	28.692
H	26.233	30.419	29.949
H	24.186	24.900	32.246
H	28.410	22.051	28.659
H	28.987	27.193	25.380
C	33.158	28.914	26.147
C	32.342	28.730	27.334
C	31.693	29.762	27.907
C	31.218	29.249	29.098
C	31.481	27.880	29.012
C	31.050	26.868	29.962
C	31.298	25.562	29.956
N	32.030	24.902	28.960
C	32.217	23.707	29.495
C	33.103	22.692	28.910
C	33.836	22.859	27.770
C	34.755	21.886	27.301
C	35.261	22.387	26.180
C	34.814	23.743	26.079
N	33.866	24.013	26.992
C	35.243	24.698	25.186
C	34.965	26.144	25.112
C	35.551	27.159	24.310
C	34.801	28.270	24.504
C	33.904	27.982	25.474
N	33.894	26.661	25.822
C	31.498	23.537	30.699
C	30.968	24.712	31.011
N	32.140	27.582	27.960
H	32.004	30.807	27.857
H	30.944	29.835	29.965
H	34.871	20.906	27.753
H	35.908	21.860	25.495
H	36.392	27.151	23.623
H	34.538	28.865	23.641
H	31.170	22.583	31.112
H	30.391	24.955	31.895
Fe	32.670	25.630	27.220
H	33.179	29.871	25.760
H	30.474	27.209	30.747
H	33.177	21.785	29.397
H	35.864	24.342	24.443

Table S9. Structure 9 XYZ Coordinates

Atom	X	Y	Z
C	30.001	27.004	31.676
C	30.846	26.187	32.549
C	31.906	26.713	33.302
C	32.384	25.634	34.063
C	31.687	24.549	33.563
C	32.064	23.150	33.729
C	31.515	22.101	33.057
N	30.550	22.269	32.079
C	30.417	20.994	31.646
C	29.596	20.580	30.530
C	28.556	21.321	30.007
C	27.721	20.945	28.883
C	26.936	21.968	28.641
C	27.282	22.991	29.569
N	28.311	22.614	30.393
C	26.720	24.212	29.639
C	27.410	25.427	30.031
C	27.399	26.675	29.314
C	28.149	27.427	30.162
C	28.874	26.649	31.008
N	28.369	25.383	31.037
C	31.480	20.166	32.182
C	32.089	20.828	33.163
N	30.684	24.902	32.764
H	32.262	27.737	33.327
H	33.145	25.758	34.829
H	27.767	20.030	28.309
H	26.198	22.078	27.848
H	26.727	27.084	28.560
H	27.670	28.326	30.539
H	31.697	19.144	31.900
H	33.040	20.543	33.608
Fe	29.131	23.704	32.067
H	30.303	27.983	31.552
H	32.808	22.938	34.412
H	29.802	19.667	30.095
H	25.720	24.290	29.395
C	29.815	30.413	26.334
C	30.157	29.071	26.817
C	31.435	28.776	27.362
C	31.520	27.388	27.379
C	30.316	26.943	26.854
C	30.039	25.521	26.497
C	29.001	25.028	25.732
N	27.954	25.788	25.306
C	27.158	24.987	24.548
C	25.821	25.370	24.061
C	25.425	26.658	23.933
C	24.176	26.940	23.314
C	24.101	28.291	23.314
C	25.254	28.772	23.879
N	26.082	27.793	24.288
C	25.571	30.080	24.046
C	26.862	30.691	24.578
C	28.745	30.794	25.613
N	27.728	29.910	25.282
C	27.708	23.695	24.598
C	28.778	23.639	25.413
N	29.482	27.994	26.564
H	32.200	29.452	27.737
H	32.396	26.796	27.624
H	23.409	26.224	23.051
H	23.189	28.890	23.297
H	27.247	22.815	24.157
H	29.484	22.826	25.340
Fe	27.637	27.918	25.739
C	27.309	32.003	24.396
C	28.510	32.004	25.063
H	26.694	32.841	24.076
H	29.331	32.600	24.660
H	30.481	31.162	26.579
H	30.703	24.822	26.866
H	25.157	24.623	23.806
H	24.838	30.754	23.776

Table S10. Structure 10 XYZ Coordinates

Atom	X	Y	Z
C	27.134	28.875	29.158
C	25.686	28.675	29.330
C	24.842	29.481	30.183
C	23.567	28.950	30.093
C	23.730	27.896	29.200
C	22.636	27.044	28.701
C	22.806	25.936	27.909
N	23.946	25.339	27.487
C	23.570	24.276	26.760
C	24.528	23.290	26.153
C	25.828	23.412	26.122
C	26.685	22.509	25.490
C	27.913	22.994	25.719
C	27.760	24.195	26.476
N	26.493	24.458	26.722
C	28.742	25.109	26.708
C	28.764	26.365	27.423
C	29.896	27.080	27.826
C	29.350	28.225	28.351
C	27.937	28.084	28.424
N	27.564	26.885	27.831
C	22.204	24.187	26.721
C	21.711	25.188	27.472
N	24.976	27.719	28.772
H	25.114	30.278	30.866
H	22.686	29.359	30.564
H	26.423	21.606	24.949
H	28.770	22.619	25.166
H	30.930	26.985	27.536
H	29.874	29.142	28.131
H	21.644	23.450	26.155
H	20.670	25.321	27.724
Fe	25.647	26.327	27.294
H	27.565	29.682	29.636
H	21.672	27.299	28.966
H	24.124	22.443	25.723
H	29.651	24.855	26.291
C	34.803	27.823	27.041
C	33.814	27.618	28.132
C	33.019	28.639	28.659
C	32.379	28.039	29.722
C	32.693	26.708	29.659
C	32.005	25.648	30.480
C	32.304	24.296	30.508
N	33.257	23.687	29.768
C	33.267	22.430	30.196
C	34.234	21.527	29.701
C	35.248	21.746	28.709
C	36.156	20.676	28.265
C	36.838	21.248	27.232
C	36.430	22.625	27.214
N	35.539	22.940	28.129
C	37.102	23.604	26.448
C	36.771	25.020	26.294
C	37.407	25.943	25.438
C	36.684	27.081	25.672
C	35.703	26.883	26.601
N	35.772	25.570	27.024
C	32.194	22.167	31.159
C	31.555	23.367	31.237
N	33.608	26.444	28.734
H	32.814	29.623	28.242
H	31.562	28.580	30.194
H	36.153	19.628	28.541
H	37.474	20.746	26.509
H	38.019	25.660	24.589
H	37.122	28.074	25.655
H	31.938	21.250	31.676
H	30.636	23.577	31.782
Fe	34.357	24.588	28.132
H	34.814	28.745	26.577
H	31.230	25.962	31.085
H	34.208	20.579	30.109
H	37.933	23.278	25.931

Table S11. Structure 11 XYZ Coordinates

Atom	X	Y	Z
C	28.060	28.725	27.898
C	26.659	28.637	28.265
C	25.943	29.603	28.958
C	24.693	29.020	29.213
C	24.672	27.836	28.498
C	23.499	27.086	28.160
C	23.516	25.807	27.778
N	24.694	25.109	27.439
C	24.204	23.921	27.064
C	25.085	22.759	26.719
C	26.426	22.739	26.696
C	27.208	21.533	26.505
C	28.496	21.949	26.595
C	28.456	23.383	26.778
N	27.206	23.845	26.946
C	29.472	24.226	26.700
C	29.526	25.694	26.996
C	30.651	26.426	27.221
C	30.182	27.672	27.304
C	28.821	27.704	27.466
N	28.352	26.451	27.240
C	22.749	23.883	27.127
C	22.308	25.096	27.618
N	25.886	27.583	27.979
H	26.159	30.617	29.272
H	23.961	29.615	29.731
H	26.876	20.499	26.369
H	29.386	21.420	26.266
H	31.703	26.158	27.207
H	30.762	28.525	26.984
H	22.157	23.022	26.838
H	21.323	25.458	27.892
Fe	26.459	25.963	26.930
H	28.511	29.650	27.975
H	22.586	27.563	28.215
H	24.608	21.878	26.472
H	30.363	23.810	26.388
C	33.679	28.675	27.736
C	32.932	28.363	28.970
C	32.124	29.243	29.602
C	31.338	28.514	30.524
C	31.688	27.219	30.337
C	31.061	26.028	30.933
C	31.673	24.830	31.049
N	32.799	24.454	30.432
C	33.059	23.263	30.891
C	34.217	22.441	30.386
C	35.045	22.750	29.284
C	35.838	21.716	28.691
C	36.639	22.424	27.759
C	36.032	23.688	27.690
N	35.028	23.912	28.574
C	36.300	24.628	26.806
C	35.802	26.072	26.667
C	36.163	27.027	25.720
C	35.367	28.093	25.984
C	34.537	27.809	27.053
N	34.669	26.461	27.404
C	32.359	22.950	32.063
C	31.350	23.840	32.033
N	32.682	27.120	29.375
H	32.179	30.333	29.624
H	30.700	28.925	31.291
H	36.039	20.773	29.193
H	37.320	21.993	27.040
H	37.045	27.005	25.092
H	34.873	28.603	25.162
H	32.364	21.960	32.515
H	30.460	23.872	32.666
Fe	33.525	25.386	28.685
H	33.556	29.620	27.341
H	30.094	26.114	31.284
H	34.425	21.568	30.895
H	36.967	24.340	26.073

Table S12. Structure 12 XYZ Coordinates

Atom	X	Y	Z
C	27.556	29.819	28.424
C	27.695	30.122	26.966
C	27.591	31.369	26.368
C	28.007	31.170	24.999
C	28.252	29.857	24.899
C	28.603	29.158	23.657
C	28.462	27.863	23.344
N	28.123	26.859	24.202
C	28.189	25.713	23.553
C	28.104	24.417	24.238
C	27.925	24.143	25.564
C	27.924	22.863	26.156
C	27.732	23.055	27.461
C	27.630	24.472	27.642
N	27.780	25.154	26.497
C	27.559	25.050	28.937
C	27.479	26.473	29.281
C	26.998	27.079	30.413
C	27.389	28.409	30.338
C	27.693	28.567	29.070
N	27.812	27.401	28.354
C	28.506	25.999	22.188
C	28.787	27.312	22.107
N	28.031	29.146	26.052
H	27.018	32.228	26.693
H	28.163	31.934	24.250
H	27.845	21.893	25.688
H	27.728	22.355	28.295
H	26.531	26.578	31.256
H	27.952	28.855	31.140
H	28.693	25.340	21.351
H	29.184	27.858	21.263
Fe	28.411	27.082	26.224
H	27.333	30.621	29.034
H	29.018	29.749	22.919
H	28.193	23.590	23.627
H	27.564	24.390	29.730
C	32.713	28.267	29.309
C	31.929	28.124	30.530
C	31.005	29.023	30.933
C	30.361	28.358	31.954
C	31.005	27.168	32.134
C	30.679	26.152	33.118
C	31.216	24.896	33.240
N	32.134	24.325	32.408
C	32.318	23.054	32.881
C	33.369	22.142	32.395
C	34.167	22.301	31.317
C	35.095	21.321	30.901
C	35.645	21.809	29.735
C	35.107	23.217	29.688
N	34.137	23.416	30.551
C	35.416	24.223	28.824
C	34.843	25.533	28.495
C	35.161	26.401	27.436
C	34.376	27.487	27.586
C	33.608	27.306	28.754
N	33.900	26.061	29.317
C	31.531	22.917	34.017
C	30.731	23.974	34.237
N	31.969	26.988	31.231
H	30.937	30.084	30.717
H	29.803	28.848	32.740
H	35.440	20.529	31.554
H	36.463	21.408	29.167
H	35.916	26.233	26.682
H	34.822	28.445	27.374
H	31.574	22.037	34.652
H	30.181	24.134	35.146
Fe	33.264	25.285	31.096
H	32.609	29.155	28.794
H	29.956	26.405	33.809
H	33.512	21.280	32.944
H	36.258	24.013	28.265

Table S13. Structure 13 XYZ Coordinates

Atom	X	Y	Z
C	28.088	29.297	29.776
C	28.495	28.360	28.797
C	29.311	28.756	27.757
C	29.293	27.659	26.882
C	28.539	26.685	27.449
C	28.142	25.488	26.733
C	27.172	24.604	27.199
N	26.496	24.741	28.367
C	25.774	23.634	28.505
C	24.803	23.367	29.562
C	24.580	24.036	30.680
C	23.702	23.758	31.748
C	23.754	24.798	32.589
C	24.837	25.681	32.144
N	25.303	25.220	30.946
C	25.153	26.904	32.706
C	25.858	28.021	32.114
C	26.088	29.250	32.675
C	26.753	29.888	31.770
C	27.211	29.012	30.775
N	26.587	27.772	30.917
C	25.895	22.781	27.295
C	26.809	23.487	26.535
N	28.048	27.114	28.639
H	30.082	29.518	27.835
H	29.924	27.561	26.001
H	23.017	22.925	31.809
H	23.140	24.870	33.473
H	25.791	29.578	33.666
H	26.489	30.901	31.516
H	25.388	21.853	27.084
H	27.070	23.176	25.533
Fe	26.947	26.010	29.880
H	28.479	30.251	29.729
H	28.602	25.281	25.833
H	24.206	22.538	29.415
H	24.844	27.039	33.681
C	32.668	29.273	27.189
C	32.933	28.398	26.053
C	33.183	28.755	24.752
C	33.554	27.553	24.080
C	33.745	26.662	25.116
C	34.592	25.483	24.988
C	34.793	24.501	25.952
N	34.202	24.495	27.176
C	34.566	23.273	27.733
C	34.017	22.856	29.088
C	33.496	23.716	30.044
C	33.133	23.281	31.323
C	32.647	24.348	31.896
C	32.629	25.431	30.973
N	33.096	25.035	29.779
C	32.199	26.712	31.274
C	32.377	27.900	30.422
C	32.277	29.302	30.752
C	32.440	29.896	29.548
C	32.594	28.931	28.540
N	32.627	27.679	29.058
C	35.402	22.534	26.816
C	35.589	23.332	25.762
N	33.181	27.109	26.264
H	32.971	29.737	24.350
H	33.707	27.390	23.022
H	33.243	22.242	31.609
H	32.401	24.380	32.947
H	32.193	29.838	31.693
H	33.180	30.678	29.433
H	35.806	21.533	26.899
H	36.230	23.123	24.912
Fe	32.973	25.913	27.991
H	32.516	30.269	26.966
H	35.092	25.365	24.093
H	34.033	21.850	29.316
H	31.711	26.846	32.174

Table S14. Structure 14 XYZ Coordinates

Atom	X	Y	Z
C	27.782	29.196	29.674
C	27.312	28.386	30.801
C	26.749	28.898	31.888
C	26.490	27.812	32.722
C	26.665	26.736	31.904
C	26.382	25.251	32.165
C	26.829	24.186	31.429
N	27.705	24.245	30.393
C	27.926	22.969	30.078
C	28.863	22.551	29.009
C	29.254	23.337	27.967
C	29.945	22.776	26.850
C	29.908	23.783	25.936
C	29.283	24.901	26.620
N	28.902	24.618	27.830
C	29.080	26.169	26.052
C	28.803	27.424	26.785
C	28.822	28.745	26.235
C	28.673	29.496	27.368
C	28.242	28.746	28.438
N	28.364	27.418	28.064
C	27.189	22.098	30.907
C	26.408	22.835	31.706
N	27.202	27.082	30.744
H	26.971	29.857	32.354
H	26.475	27.802	33.803
H	30.166	21.715	26.748
H	30.192	23.766	24.891
H	29.049	29.218	25.296
H	29.540	30.106	27.608
H	27.101	21.022	30.853
H	25.720	22.306	32.367
Fe	27.749	25.848	29.060
H	27.771	30.219	29.812
H	25.792	25.031	32.982
H	29.248	21.595	29.057
H	29.129	26.232	25.023
C	32.506	28.926	26.931
C	32.061	28.334	28.204
C	31.669	29.051	29.273
C	31.766	28.140	30.323
C	31.886	26.917	29.674
C	32.088	25.681	30.460
C	32.468	24.435	29.977
N	32.684	24.119	28.659
C	32.869	22.758	28.620
C	33.228	22.042	27.379
C	33.434	22.654	26.175
C	33.831	21.989	25.034
C	33.841	22.877	24.017
C	33.579	24.189	24.628
N	33.284	24.029	25.941
C	33.618	25.404	23.966
C	33.513	26.683	24.522
C	33.782	27.898	23.843
C	33.417	28.825	24.681
C	32.923	28.235	25.829
N	33.110	26.849	25.816
C	32.827	22.244	29.967
C	32.538	23.307	30.821
N	32.054	27.008	28.387
H	31.472	30.092	29.511
H	31.683	28.372	31.379
H	33.792	20.919	24.889
H	34.223	22.634	23.033
H	34.048	28.025	22.794
H	34.129	29.635	24.721
H	33.205	21.292	30.339
H	32.222	23.301	31.854
Fe	32.325	25.390	27.081
H	32.502	29.956	26.866
H	31.930	25.751	31.477
H	33.330	21.016	27.424
H	33.739	25.361	22.942

Table S15. Structure 15 XYZ Coordinates

Atom	X	Y	Z
C	28.630	28.771	31.038
C	28.024	28.025	32.170
C	28.455	28.139	33.486
C	27.642	27.274	34.255
C	26.808	26.702	33.295
C	25.919	25.606	33.582
C	25.367	24.764	32.681
N	25.576	24.833	31.328
C	24.955	23.818	30.823
C	24.738	23.587	29.373
C	25.038	24.363	28.307
C	24.626	24.237	26.934
C	24.985	25.378	26.266
C	25.689	26.124	27.259
N	25.755	25.511	28.434
C	26.261	27.408	27.078
C	27.273	28.044	27.939
C	28.260	28.993	27.541
C	29.043	29.207	28.651
C	28.392	28.580	29.753
N	27.339	27.800	29.242
C	24.482	22.994	31.898
C	24.692	23.627	33.073
N	27.082	27.156	32.069
H	29.027	28.955	33.914
H	27.699	27.290	35.337
H	23.947	23.459	26.615
H	24.716	25.614	25.245
H	28.459	29.479	26.589
H	30.125	29.144	28.581
H	23.779	22.184	31.707
H	24.501	23.242	34.072
Fe	26.054	26.500	30.352
H	29.305	29.513	31.280
H	25.680	25.448	34.573
H	24.285	22.689	29.141
H	25.937	27.951	26.263
C	32.385	27.658	27.494
C	31.908	26.603	28.350
C	32.288	26.436	29.680
C	31.626	25.282	29.979
C	30.691	25.018	28.946
C	29.667	24.002	28.948
C	28.880	23.690	27.953
N	28.879	24.166	26.672
C	28.020	23.440	25.982
C	27.868	23.648	24.526
C	28.280	24.745	23.766
C	28.152	24.833	22.362
C	28.754	25.957	21.957
C	29.232	26.622	23.186
N	28.983	25.837	24.291
C	30.022	27.698	23.213
C	30.820	28.144	24.343
C	31.583	29.369	24.403
C	32.394	29.164	25.529
C	31.955	28.057	26.197
N	31.042	27.393	25.458
C	27.421	22.490	26.862
C	27.980	22.617	28.061
N	30.925	25.791	27.948
H	32.795	27.058	30.408
H	31.714	24.712	30.898
H	27.637	24.107	21.735
H	28.904	26.156	20.907
H	31.580	30.152	23.656
H	33.448	29.227	25.269
H	26.854	21.679	26.413
H	27.811	22.078	28.991
Fe	30.172	25.634	25.917
H	33.163	28.214	27.883
H	29.544	23.468	29.823
H	27.400	22.889	24.007
H	30.071	28.265	22.352
